# Chromosome map of the Siamese cobra: did partial synteny of sex chromosomes in the amniote represent “a hypothetical ancestral super-sex chromosome” or random distribution?

**DOI:** 10.1186/s12864-018-5293-6

**Published:** 2018-12-17

**Authors:** Worapong Singchat, Rebecca E. O’Connor, Panupong Tawichasri, Aorarat Suntronpong, Siwapech Sillapaprayoon, Sunutcha Suntrarachun, Narongrit Muangmai, Sudarath Baicharoen, Surin Peyachoknagul, Lawan Chanhome, Darren Griffin, Kornsorn Srikulnath

**Affiliations:** 1 Laboratory of Animal Cytogenetics and Comparative Genomics (ACCG), Department of Genetics, Faculty of Science, Kasetsart University, Bangkok, 10900 Thailand; 2 Animal Breeding and Genetics Consortium of Kasetsart University (ABG-KU), Bangkok, 10900 Thailand; 3 School of Biosciences, University of Kent, Canterbury, CT2 7NY United Kingdom; 4 Department of Research and Development, Queen Saovabha Memorial Institute, The Thai Red Cross Society, Bangkok, 10330 Thailand; 5 Department of Fishery Biology, Faculty of Fisheries, Kasetsart University, Bangkok, 10900 Thailand; 6 Bureau of Conservation and Research, Zoological Park Organization under the Royal Patronage of His Majesty the King, Bangkok, Thailand; 7 Department of Biology, Faculty of Science, Naresuan University, Phitsanulok, 65000 Thailand; 8 Snake Farm, Queen Saovabha Memorial Institute, The Thai Red Cross Society, Bangkok, 10330 Thailand; 9 Center for Advanced Studies in Tropical Natural Resources, National Research University-Kasetsart University, Kasetsart University, Bangkok, 10900 Thailand; 10 Center of Excellence on Agricultural Biotechnology (AG-BIO/PERDO-CHE), Bangkok, 10900 Thailand

**Keywords:** amniote, synteny, repeat sequences, sex chromosome, snake

## Abstract

**Background:**

Unlike the chromosome constitution of most snakes (2n=36), the cobra karyotype shows a diploid chromosome number of 38 with a highly heterochromatic W chromosome and a large morphologically different chromosome 2. To investigate the process of sex chromosome differentiation and evolution between cobras, most snakes, and other amniotes, we constructed a chromosome map of the Siamese cobra (*Naja kaouthia*) with 43 bacterial artificial chromosomes (BACs) derived from the chicken and zebra finch libraries using the fluorescence *in situ* hybridization (FISH) technique, and compared it with those of the chicken, the zebra finch, and other amniotes.

**Results:**

We produced a detailed chromosome map of the Siamese cobra genome, focusing on chromosome 2 and sex chromosomes. Synteny of the Siamese cobra chromosome 2 (NKA2) and NKAZ were highly conserved among snakes and other squamate reptiles, except for intrachromosomal rearrangements occurring in NKA2. Interestingly, twelve BACs that had partial homology with sex chromosomes of several amniotes were mapped on the heterochromatic NKAW as hybridization signals such as repeat sequences. Sequence analysis showed that most of these BACs contained high proportions of transposable elements. In addition, hybridization signals of telomeric repeat (TTAGGG)_n_ and six microsatellite repeat motifs ((AAGG)_8_, (AGAT)_8_, (AAAC)_8_, (ACAG)_8_, (AATC)_8_, and (AAAAT)_6_) were observed on NKAW, and most of these were also found on other amniote sex chromosomes.

**Conclusions:**

The frequent amplification of repeats might involve heterochromatinization and promote sex chromosome differentiation in the Siamese cobra W sex chromosome. Repeat sequences are also shared among amniote sex chromosomes, which supports the hypothesis of an ancestral super-sex chromosome with overlaps of partial syntenies. Alternatively, amplification of microsatellite repeat motifs could have occurred independently in each lineage, representing convergent sex chromosomal differentiation among amniote sex chromosomes.

**Electronic supplementary material:**

The online version of this article (10.1186/s12864-018-5293-6) contains supplementary material, which is available to authorized users.

## Background

Sex chromosomes evolved independently from a homologous autosomal pair in both plants and animals when one chromosomal partner acquired a sex-determining allele [1, 2]. Differentiated sex chromosomes then developed from the accumulation of sexually antagonistic alleles at loci linked to the sex-specific region. To maintain linkage of these genes, meiotic recombination between proto-sex chromosomes was suppressed around the heterologous region in one sex, which in turn promoted the loss of active genes or segmental insertions and deletions, and caused this region to extend along part or all of the chromosome. This produced chromosomal morphological differences between the X and Y (male heterogamety) or Z and W (female heterogamety) sex chromosomes. It has also been proposed that structural changes in sex chromosomes might expedite the suppression of recombination, favoring the further amplification of repeat sequences and leading to heterochromatinization [3]. It is thus of great importance to understand the mechanism and role of sex chromosome differentiation with regard to sex determination.

Sex chromosomes of amniotes are highly diverse, ranging from cryptic to highly heteromorphic with XY or ZW systems. Using comparative gene mapping (chromosome mapping via a cytogenetic technique) and whole-genome sequencing, genomic convergence was discovered, in which unrelated sex chromosomes share syntenies across distantly related taxa [4]. Highly conserved synteny of the chicken Z chromosome has been found in the X chromosomes of the Mexican giant musk turtle (*Staurotypus triporcatus*) and the giant musk turtle (*S. salvinii*) [5]. However, the X chromosomes of the marsh turtle (*Siebenrockiella crassicollis*) and wood turtle (*Glyptemys insculpta*) correspond to chicken chromosome 5, while the Z chromosome of the Chinese softshell turtle (*Pelodiscus sinensis*) and the spiny softshell turtle (*Apalone spinifera*) corresponds to chicken chromosome 15 [6–9]. The Hokou gecko (*Gekko hokouenesis*, Gekkota) Z chromosome is homologous with the chicken Z chromosome [10, 11], whereas the micro-X chromosome of the Anolis lizard (*Anolis carolinensis*, Iguania), the micro-Z chromosome of the dragon lizard (*Pogona vitticeps*, Iguania), and the Z chromosome of the sand lizard (*Lacerta agilis*, Lacertoidea) have homology with chicken chromosomes 15, 17 and 23, and 6 and 9, respectively [4, 12–14]. Notably, monotreme sex chromosomes also share partial synteny with the chicken Z chromosome [15], which corresponds partially to squamate chromosome 2. Comparative genomics, based on chromosome painting and gene mapping for several squamate reptiles, have revealed that the chicken Z chromosome is homologous with the short arm of the bi-armed chromosome 2 in most squamate reptiles [11, 13, 14, 16–19]. This suggests the possibility that squamate reptile chromosome 2 is part of a larger ancestral amniote super-sex chromosome with overlaps of partial syntenies among amniotes. However, the hypothesis of an ancestral super-sex chromosome does not just relate to the homology of chicken Z chromosome, but states that unrelated sex chromosomes share syntenies across distantly related taxa. Ancestral super-sex chromosomes probably exist in amniotes. Multiple translocations have led to the appearance of sauropsid and diapsid sex chromosomes [4].

Snakes (Serpentes), a species-rich lineage of extant reptiles, exhibit conserved ZZ/ZW-type sex chromosomes in most species, except for *Boa imperator*, *Python bivittatus*, *P. regius* [20–24]. The Z chromosome, which is similar in size across species, is the fourth or fifth largest metacentric chromosome, whereas the W chromosome varies from being homomorphic in most henophidians (primitive snakes) to highly differentiated in caenophidians (advanced snakes), as a consequence of different centromere positions and/or amounts of heterochromatin [25–31]. Large amplification of microsatellite repeat motifs or telomeric repeats have been found in the W chromosome of several colubroid snakes [25, 26], and genomic comparisons have revealed that syntenies with the snake sex chromosome are also present in a large variety of chromosomes in squamate reptiles [4, 11–14, 16–18, 32–34]. However, the chicken Z chromosome is homologous with the short arm of snake chromosome 2, as also found in other squamate reptiles, while the snake Z chromosome is homologous with chicken chromosomes 2 and 27 [21–24, 32–34]. Interestingly, the non-homologous W chromosomes of chickens and the common tiger snake (*Notechis scutatus*) share common repeat sequences that are not present elsewhere in the genomes analyzed to date [30]. This suggests that repeat sequences are partially shared between the sex chromosomes of the chicken and snake, and supports the hypothesis that squamate chromosome 2 is part of an ancestral super-sex chromosome in amniotes [4].

Unlike most snakes, which have a chromosome number of 2n=36, consisting of 16 macrochromosomes and 20 microchromosomes [29, 35], cobra (*Naja* spp.) karyotypes show 2n=38, comprising five pairs of macrochromosomes and 14 pairs of microchromosomes [35]. In the Siamese cobra (*Naja kaouthia*), the W chromosome exhibits highly remarkable amplified telomeric repeats, indicating the role of repeat sequences for sex chromosome differentiation. Whereas most snakes have a large submetacentric chromosome 2, which is also highly conserved in other squamate reptiles, cobras have a large subtelocentric chromosome 2 [35]. Examination of cobra karyotypes points to several fusions and fissions in the *Naja* lineage, suggesting that the subtelocentric chromosome 2 in the *Naja* lineage appeared by centric fission of a large submetacentric chromosome 2 in ancestral snake karyotypes. By contrast, examples of chromosome 2 resembling those of *Naja* are not found in the majority of squamate reptiles; however, several examples of lizard subtelocentric/acrocentric chromosome 2 show homology with chromosome 2 of most squamate reptiles [11, 14, 16–19]. An alternative explanation should be considered, whereby the subtelocentric chromosome 2 appeared by intrachromosomal rearrangements in the *Naja* lineage, although no reports have confirmed this. With these two possible scenarios involving the W chromosome and structural change in snake chromosome 2, cobras are very good models with which to study the process of sex chromosome differentiation and evolution, including the hypothesis of an ancestral super-sex chromosome in amniotes. In this study, we constructed a comparative chromosome map with 43 bacterial artificial chromosomes (BACs) derived from the chicken and zebra finch genomes using fluorescence *in situ* hybridization (FISH) [36–38], and compared the chromosome homology of the Siamese cobra (*N. kaouthia*) with those of the Japanese striped snake (*Elaphe quadrivirgata*) and other amniotes. We also screened the Siamese cobra chromosomes using FISH with 19 microsatellite repeat motifs and determined *in silico* the copy number of microsatellite repeats on the chicken and zebra finch BAC sequences for comparison. This allowed us to delineate the synteny between species, to investigate its significance, and to discuss the hypothesis of an ancestral super-sex chromosome in amniotes.

## Results

### Karyotype and Chromosomal Locations of Telomeric Repeat and Microsatellite Repeat Motifs

**Fig. 1:**
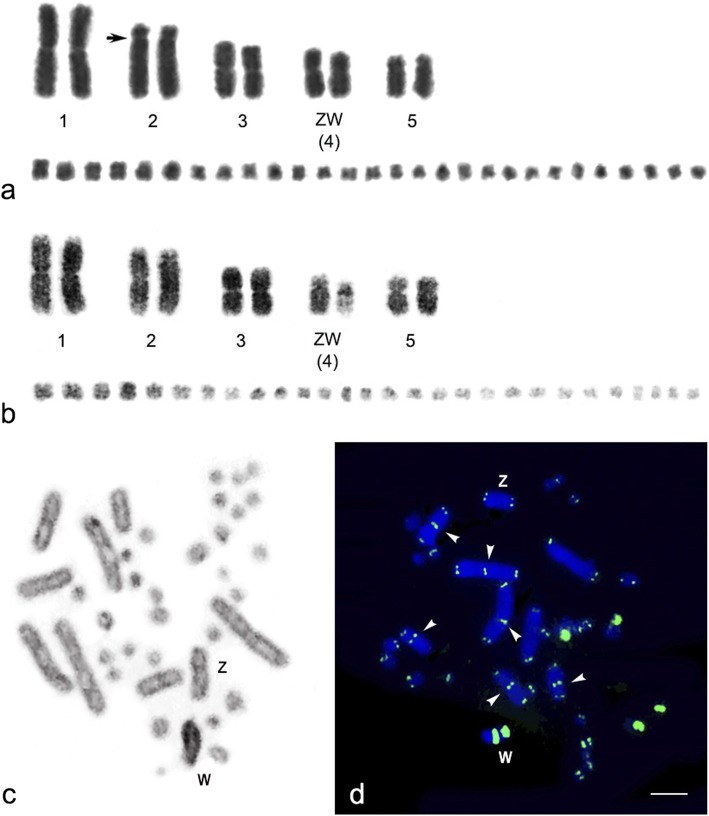
Karyotype and metaphase spread of the Siamese cobra (*Naja kaouthia*). Giemsa- (**a**), grey images of DAPI-stained karyotypes (**b**), and C-banded metaphase spread (**c**) of female Siamese cobra (*N. kaouthia*). FISH patterns of the telomeric (TTAGGG)_n_ sequence on DAPI-stained metaphase chromosome spreads of female Siamese cobra (*N. kaouthia*) (**d**). Arrows indicate the centromeric region of chromosome 2 and arrowheads indicate signals of interstitial telomeric sites. “Z” indicates Z sex chromosome, and “W” indicates W sex chromosome. Scale bar represents 10 μm

**Fig. 2:**
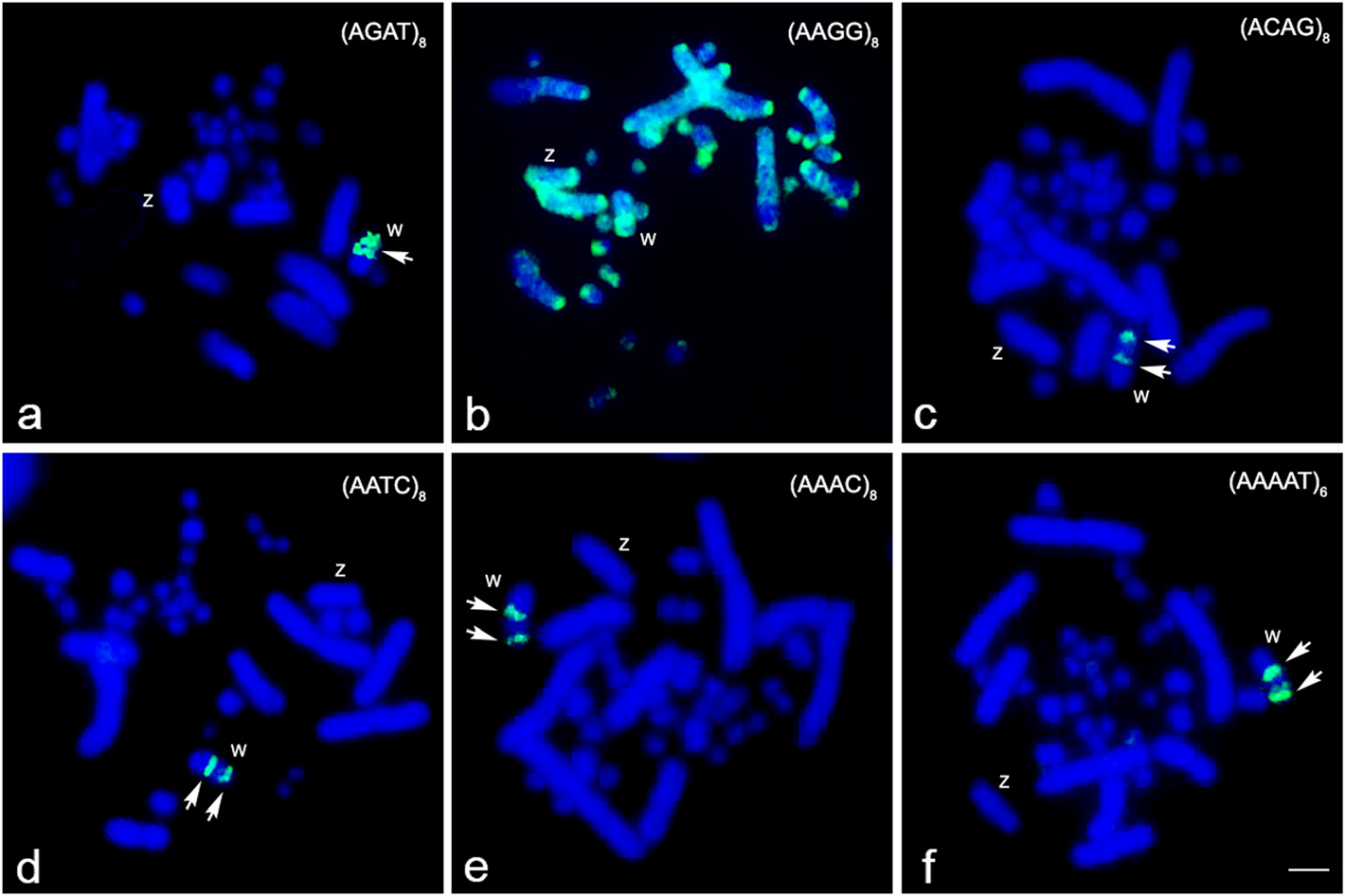
Chromosomal locations of microsatellite repeat motifs in the Siamese cobra (*Naja kaouthia*). Hybridization patterns of FITC-labeled (AGAT)_8_ (**a**), (AAGG)_8_ (**b**), (ACAG)_8_ (**c**), (AATC)_8_ (**d**), (AAAC)_8_ (**e**), and (AAAAT)_6_ (**f**) on DAPI-stained chromosomes. Arrows indicate the hybridization signals. “Z” indicates Z sex chromosome, and “W” indicates W sex chromosome. Scale bar represents 10 μm

We examined more than 20 DAPI-stained metaphase spreads from two males and two females. Chromosome numbers were 2n=38, comprising 10 macrochromosomes and 28 microchromosomes in both sexes. The macrochromosomes comprised one pair of large metacentrics (first), one pair of large subtelocentrics (second), two pairs of medium-sized metacentrics (third and fourth), and one pair of submetacentric (fifth). The fourth largest chromosomes were sex chromosomes, in which the female karyotype contained the metacentric Z chromosome and the submetacentric W chromosome (Figs. 1a and b). The DAPI-stained chromosome banding pattern showed large DAPI-negative bands were on the long arm of the W chromosome, indicating GC richness in this region. C-banding revealed that the W chromosome exhibited C-positive heterochromatin in the entire region (Fig. 1c). Hybridization signals of hexamer repeat sequences (TTAGGG)_n_ were found at the ends of all chromosomes. High intensity signals were observed on two microchromosome pairs. Interstitial telomeric sites were found on three macrochromosome pairs in either sex, while two large-sized (TTAGGG)_n_ signals on the q arm of the W chromosome were also observed, but not on the Z chromosome (Fig. 1d). Eleven of nineteen microsatellite repeat motifs were successfully mapped onto most *N. kaouthia* chromosomes (NKA) (Fig. 2, Additional file 1: Figure S1); however, only six microsatellite repeat motifs showed high amplification on the W chromosome. Strong hybridization signals of tetranucleotide (AAGG)_8_ were localized to NKAWq in addition to the predominant telomeric sites of most chromosomes. By contrast, (AGAT)_8_, (AAAC)_8_, (ACAG)_8_, (AATC)_8_, and (AAAAT)_6_ repeat motifs were specifically mapped to NKAWq (Fig. 2).

### Chromosome Homology among the Siamese cobra, Chicken, and Zebra finch

**Fig. 3:**
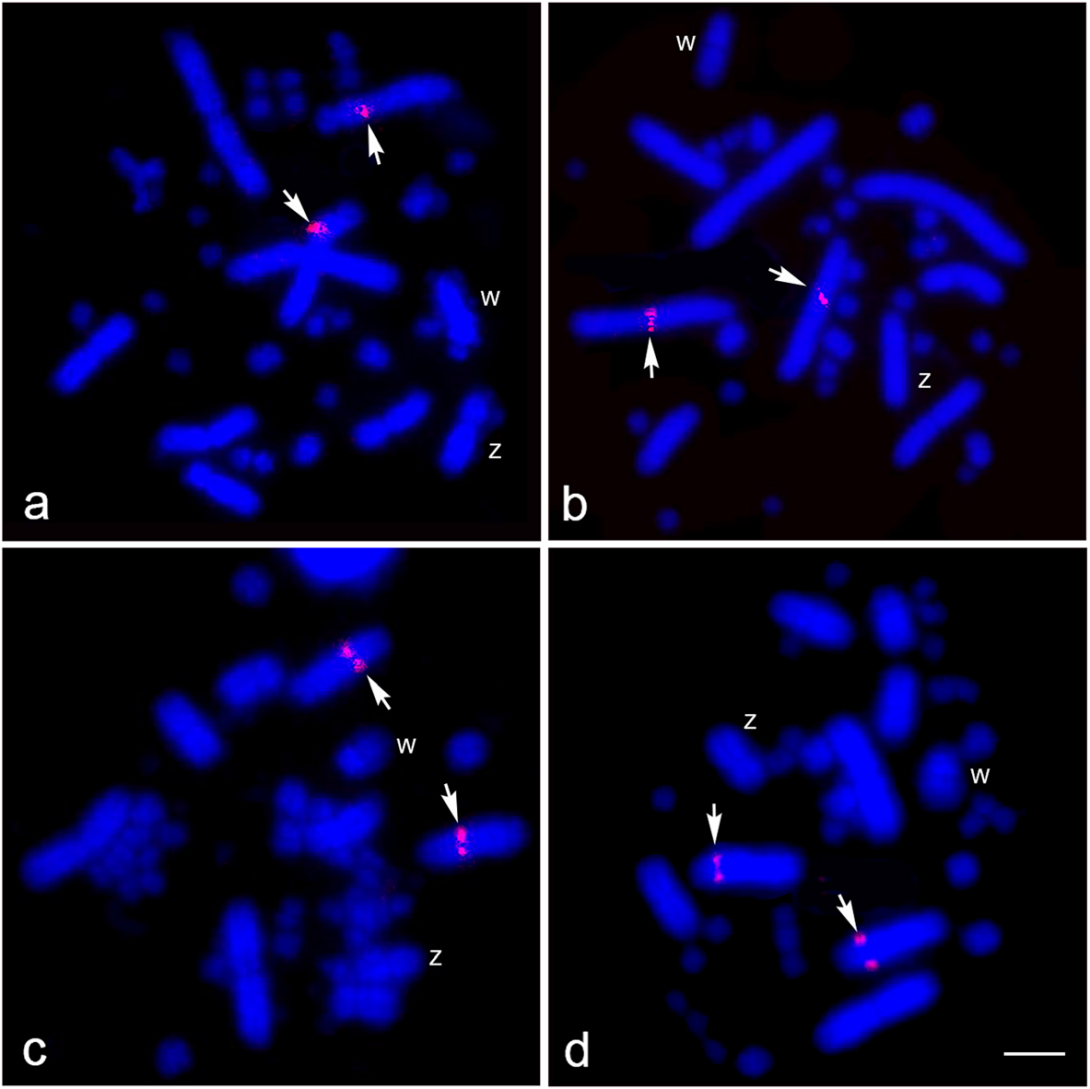
Chromosomal locations of chicken and zebra finch BACs in female Siamese cobra (*Naja kaouthia*). GGAZ BAC (CH261-133M4) (**a**), GGA18 BAC (CH261-67N15) (**b**), GGA18 BAC (CH261-72B18) (**c**), and GGA12 BAC (TGMCBA-305E19) (**d**) were localized to chromosome 2 (NKA2). Arrows indicate the hybridization signals. “Z” indicates Z sex chromosome, and “W” indicates W sex chromosome. Scale bar represents 10 μm

**Fig. 4:**
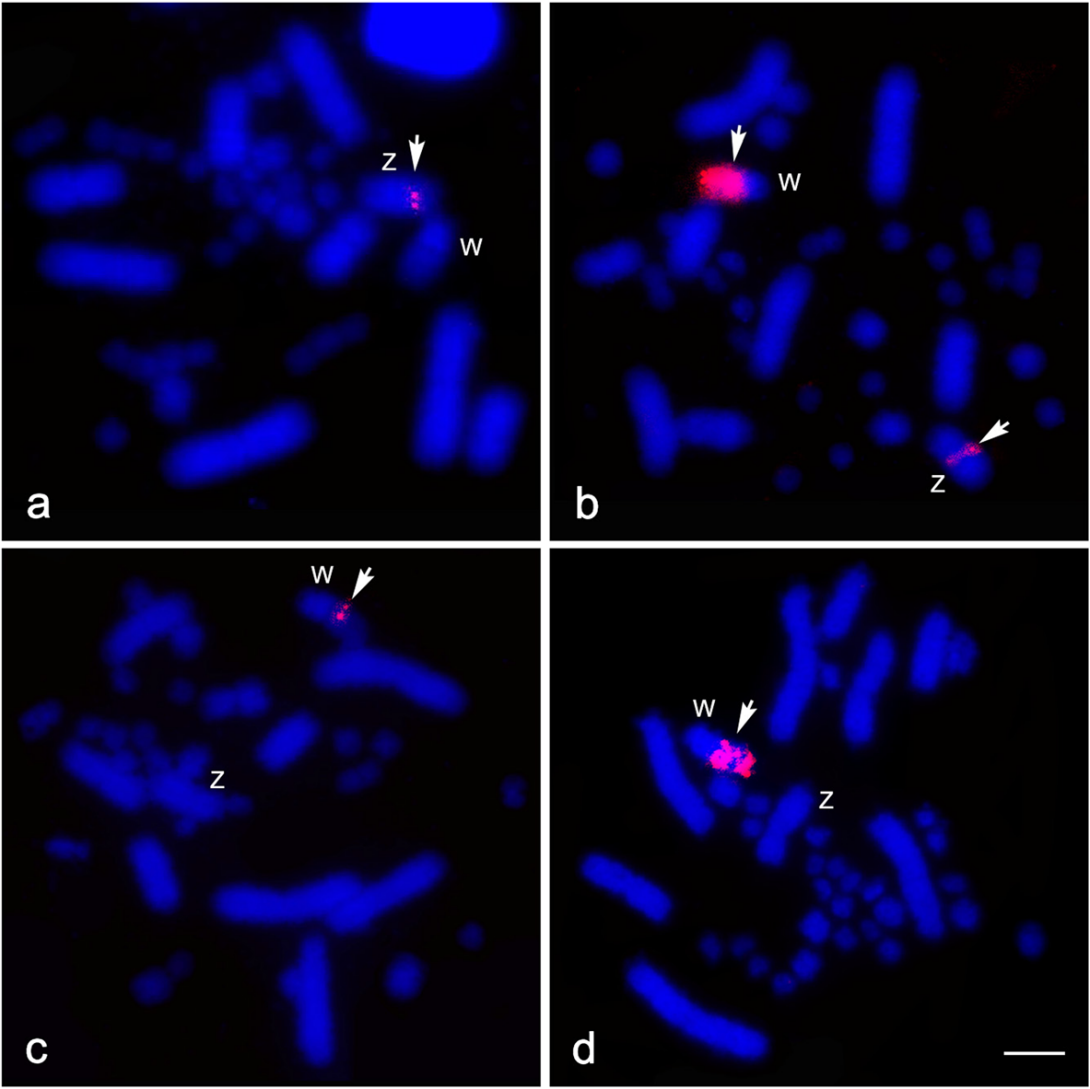
Chromosomal locations of chicken and zebra finch BAC clones in female Siamese cobra (*Naja kaouthia*). GGA2p BAC (CH261-177K1) (**a**), TGU27 BAC (TGMCBA-23C5) (**b**), GGA9 BAC (CH261-95N3) (**c**), and GGAZ BAC (CH261-129A16) (**d**) were localized to W chromosome (NKAW). Arrows indicate the hybridization signals. “Z” indicates Z sex chromosome, and “W” indicates W sex chromosome. Scale bar represents 10 μm

Twenty-six chicken BACs and seventeen zebra finch BACs were mapped to the Siamese cobra chromosomes. We constructed a chromosome map for the Siamese cobra with 43 BACs (markers) (Table 1, Figs. 3 and 4, Additional file 2: Figure S2). More than 20 metaphase spreads were observed for each BAC, with hybridization efficiencies ranging from approximately 70% to 90%. Chromosome homology between *N. kaouthia*, chicken and zebra finch was analyzed using the chicken genome database (http://www.ncbi.nlm.nih.gov/genome/guide/chicken/) and zebra finch genome database (http://www.ncbi.nlm.nih.gov/genome/367). Seven BACs mapped to NKA2 were localized to GGA1, GGA18 and GGAZ and TGU12 and TGUZ. Three BACs mapped to NKAZ were localized to GGA2p, GGA27, and TGU27. However, twelve BACs on NKAW were located on GGA1, GGA4, GGA9, GGAZ, TGU1B, TGU9, TGU13, TGU17, TGU23, TGU27, and TGUZ. The remaining 22 BACs on NKA1 and microchromosomes were located on GGA1, GGA4, GGA5, GGA9, GGA15, GGA17, GGA23, TGU4A, TGU5, TGU15 and TGU23 (Table 1).

**Table 1:** List of chicken and zebra finch BACs mapped to the Siamese cobra chromosomes and their chromosomal location in the chicken (*Gallus gallus*)

Number	Chicken chromosome	BAC	Chromosomal location in *Naja kaouthia*
1	1	CH261-89C18	Wq
2	1	CH261-184E5	micro
3	1	CH261-125F1	2q
4	1	TGMCBA-167P13	Wq
5	1	CH261-36B5	micro
6	1	CH261-18J16	micro
7	1	CH261-83O13	micro
8	2p	CH261-177K1	Z
9	4	CH261-71L6	Wq
10	4	CH261-18C6	1q and micro
11	4	CH261-85H10	micro
12	4	TGMCBA-200G5	micro
13	4	TGMCBA-280M7	micro
14	4	TGMCBA-330J11	micro
15	5	CH261-2I23	1q
16	5	TGMCBA-145C6	1q
17	5	CH261-49B22	1q and micro
18	9	CH261-95N3	Wq
19	9	TGMCBA-217A3	Wq
20	9	CH261-183N19	micro
21	9	CH261-187M16	micro
22	12	TGMCBA-305E19	2q
23	13	TGMCBA-136I12	Wq
24	15	CH261-90P23	micro
25	15	TGMCBA-266G23	micro
26	17	TGMCBA-375I5	Wq
27	17	TGMCBA-67H23	Wq
28	17	CH261-69M11	micro
29	18	CH261-60N6	2q
30	18	CH261-67N15	2q
31	18	CH261-72B18	2q
32	23	TGMCBA-227A15	Wq
33	23	CH261-191G17	micro
34	23	CH261-105P1	micro
35	23	CH261-49G9	micro
36	23	TGMCBA-173N15	micro
37	23	TGMCBA-48O8	micro
38	27	TGMCBA-23C5	Zq and Wq
39	27	CH261-66M16	Z
40	Z	CH261-129A16	Wq
41	Z	TGMCBA-200J22	Wq
42	Z	TGMCBA-270I9	2q
43	Z	CH261-133M4	2q

### Sequence Analysis of Chicken and Zebra finch BACs Located on the Siamese cobra W sex chromosomes

The content of repeat sequences in chicken and zebra finch BACs mapped to NKAW was examined using Repbase-GIRI and MSATCOMMANDER (Tables 2 and 3). All BACs contained 3.04–24.85% repeat sequences, of which 0.07–1.26% were transposable elements. The BACs mapped to NKAW tended to have twice as many repeat sequences as BACs mapped to NKAZ, NKA2, or other chromosomes (Additional files 3, 4, 5, 6, 7 and 8: Tables S1, S2, S3, S4, S5, and S6), showing no preferential accumulation of any specific repeat sequences. Our analysis identified several microsatellite repeat motifs within BACs mapped to NKAW, most of which were not the same as those found using microsatellite repeat motif mapping (Table 3, Fig. 2). Additionally, BLAST analysis of BAC sequences mapped to NKAW revealed the predictions of five functional genes, including *DDX26B*, *DHX36*, *FHL1*, *GPR149*, and *MAP7D3,* which were related to sex determination and sex development in vertebrates (Table 4).

**Table 2:** Comparison of major classes of repeat sequences in chicken and zebra finch BACs mapped on the Siamese cobra W chromosome

	NE	LO	%	NE	LO	%	NE	LO	%	NE	LO	%
	1 CH261-89C18			1 TGMCBA-167P13			4 CH261-71L6			9 CH261-95N3		
%GC			47.61			59.52			44.85			42.81
Satellites	2	580	0.34	1	19	0.02	0	0	0	0	0	0
Simple repeats	30	988	0.58	23	2011	1.91	36	1910	1.02	70	2777	1.43
Retroelement	94	16874	9.85	37	11027	10.47	21	3668	1.95	17	3847	1.99
1) SINEs	1	49	0.03	0	0	0	2	207	0.11	2	353	0.18
2) LINEs	92	16749	9.77	33	9768	9.28	19	3461	1.84	13	2849	1.47
3) LTR elements	1	76	0.04	4	1259	1.20	0	0	0	2	645	0.33
DNA transposons	4	203	0.12	0	0	0	5	137	0.07	8	1014	0.52
Unclassified	0	0	0	0	0	0	0	0	0	2	330	0.17
Total interspersed repeats		17077	9.97		5191	2.68		3805	2.02		5191	2.68
	9 TGMCBA-217A3			13 TGMCBA-136I12			17 TGMCBA-375I5			17 TGMCBA-67H23		
%GC			48.72			51.96			44.44			51.09
Satellites	1	260	0.12	0	0	0	0	0	0	2	1074	0.64
Simple repeats	65	3487	1.63	87	6437	3.8	41	1769	1.24	45	2772	1.66
Retroelement	24	5688	2.66	51	11247	6.64	62	19590	13.73	44	11042	6.6
1) SINEs	0	0	0	1	90	0.05	2	183	0.13	0	0	0
2) LINEs	13	2373	1.11	34	5458	3.22	42	11979	8.4	32	6943	4.15
3) LTR elements	11	3315	1.55	16	5699	3.37	18	7428	5.21	12	4099	2.45
DNA transposons	8	815	0.24	16	911	0.54	6	446	0.31	7	709	0.42
Unclassified	0	0	0	3	316	0.19	1	97	0.07	0	0	0
Total interspersed repeats		6206	2.9		12474	7.37		20133	14.11		11751	7.03
	23 TGMCBA-227A15			27 TGMCBA-23C5			Z CH261-129A16			Z TGMCBA-200J22		
%GC			49.38			48.70			41.03			34.62
Satellites	0	0	0	0	0	0	1	98	0.04	0	0	0
Simple repeats	18	909	1.59	40	2247	1.11	68	2973	1.32	47	2450	1.45
Retroelement	28	7991	14	161	47194	23.26	53	26179	11.59	46	16566	9.84
1) SINEs	0	0	0	2	139	0.07	1	95	0.04	1	137	0.08
2) LINEs	15	3180	5.57	117	25323	12.48	50	25215	11.16	32	11149	6.62
3) LTR elements	13	4811	8.43	42	21732	10.71	2	869	0.38	13	5280	3.14
DNA transposons	1	85	0.15	6	979	0.48	12	2842	1.26	11	714	0.42
Unclassified	0	0	0	0	0	0	0	0	0	0	0	0
Total interspersed repeats		8076	14.15		48173	23.74		29021	12.85		17280	10.26

NE; number of elements

LO; length occupied

%; sequence percentage

**Table 3:** Comparison of frequencies of microsatellite repeat motifs in chicken and zebra finch BACs mapped on the Siamese cobra W chromosome

Chicken chromosome	BAC	Size (bp)	Number of repeats	Top five repeat motifs														
				type	bp	%	type	bp	%	type	bp	%	type	bp	%	type	bp	%
1	CH261-89C18	171,359	18	(CCATT)_11_	55	0.0321	(AGGGT)_9_	45	0.0263	(AAAC)_7_	28	0.0163	(ATC)_9_	27	0.0158	(AAC)_8_	24	0.0140
1	TGMCBA-167P13	105,288	11	(ATCC)_34_	136	0.1292	(ATC)_16_	48	0.0456	(AGC)_12_	36	0.0342	(CCT)_9_	27	0.0256	-	-	-
4	CH261-71L6	187,959	25	(CCTTT)_70_	350	0.1862	(CCTCT)_30_	150	0.0798	(GCT)_12_	36	0.0192	(GTT)_12_	36	0.0192	(AG)_17_	34	0.0181
9	CH261-95N3	193,574	31	(CCTCT)_24_	120	0.0620	(AT)_42_	84	0.0434	(AC)_30_	60	0.0310	(ATT)_18_	54	0.0279	(AAAT)_10_	40	0.0207
9	TGMCBA-217A3	214,179	41	(AGC)_32_	96	0.0448	(ACGGC)_17_	85	0.0397	(AGCCC)_14_	70	0.0327	(AT)_35_	70	0.0327	(GCT)_22_	66	0.0308
13	TGMCBA-136I12	169,361	56	(GGAT)_36_	144	0.0850	(GCT)_30_	90	0.0531	(AGC)_26_	78	0.0461	(AT)_31_	62	0.0366	(GGGCT)_12_	60	0.0354
17	TGMCBA-375I5	142,672	22	(GGAT)_54_	216	0.1514	(CCTCT)_18_	90	0.0631	(ATCC)_20_	80	0.0561	(ATT)_13_	39	0.0273	(GT)_13_	26	0.0182
17	TGMCBA-67H23	167,209	24	(AT)_54_	108	0.0646	(ATTTT)_17_	85	0.0508	(ATCCCC)_12_	72	0.0431	(GGAT)_10_	40	0.0239	(AGG)_10_	30	0.0179
23	TGMCBA-227A15	57,070	11	(AT)_16_	32	0.0561	(ATATTT)_5_	30	0.0526	(ATGT)_7_	28	0.0491	(ATTT)_6_	24	0.0421	(CCT)_8_	24	0.0421
27	TGMCBA-23C5	202,884	33	(GGAT)_43_	172	0.0848	(AAACCT)_11_	66	0.0325	(CCCTT)_9_	45	0.0222	(ATCC)_10_	40	0.0197	(AGC)_12_	36	0.0177
Z	CH261-129A16	225,875	34	(CCTCT)_70_	350	0.1550	(CTCTT)_31_	155	0.0686	(AT)_47_	94	0.0416	(CTTTTT)_12_	72	0.0319	(AAGG)_13_	52	0.0230
Z	TGMCBA-200J22	168,414	26	(CTTTTT)_24_	144	0.0855	(AAG)_34_	102	0.0606	(AAGGG)_17_	85	0.0505	(AT)_38_	76	0.0451	(AAGAGG)_12_	72	0.0428

**Table 4:** Gene list relating to sex determination and sex developmental pathways for chicken and zebra finch BACs mapped on the Siamese cobra W chromosome

BAC	Gene
CH261-89C18	–
TGMCBA-167P13	–
CH261-71L6	*DDX26B*; gene on the X-chromosome with sex-bias in multiple tissues
	*FHL1 MAP7D3*; higher levels of piRNA expression in testis than in ovary and ovotestis
CH261-95N3	–
TGMCBA-217A3	*GPR149*; novel oocyte-enriched gene, G-protein receptor 149
	*DHX36*; genes may be involved in sex development, spermatogenesis and male reproduction
TGMCBA-136I12	–
TGMCBA-375I5	–
TGMCBA-67H23	–
TGMCBA-227A15	–
TGMCBA-23C5	–
CH261-129A16	–
TGMCBA-200J22	–

– : No data

## Discussion

Comparison of the draft genome assembly and chromosome maps among amniotes revealed a high level of conservation for synteny and allows us to deduce the process of chromosomal rearrangement over millions of years [11–14, 16–18, 21, 33, 34]. However, sex chromosomes are a more dynamic entity in the genome than autosomes [3, 39]. The W chromosome of snakes is the most variable element in the snake genome, with differences in the number of genes and amplification of several repeat motifs, even between closely related species [21, 22, 25, 26]. This suggests that molecular differentiation has often occurred on the W chromosome in snakes, after divergence from a common ancestor. Amplification of repeat sequences might also have resulted in the large size of the W chromosome found in the tiger snake (*N. scutatus*) and the tiger keelback snake (*Rhabdophis tigrinus*), arising from a recent addition of repeats [26, 30, 40]. Conversely, the small size of the W chromosome in the Japanese striped snake (*E. quadrivirgata*) may be a consequence of degradation [21].

### Amplification of Microsatellite Repeat Motifs on the Siamese cobra W Chromosome

**Fig. 5:**
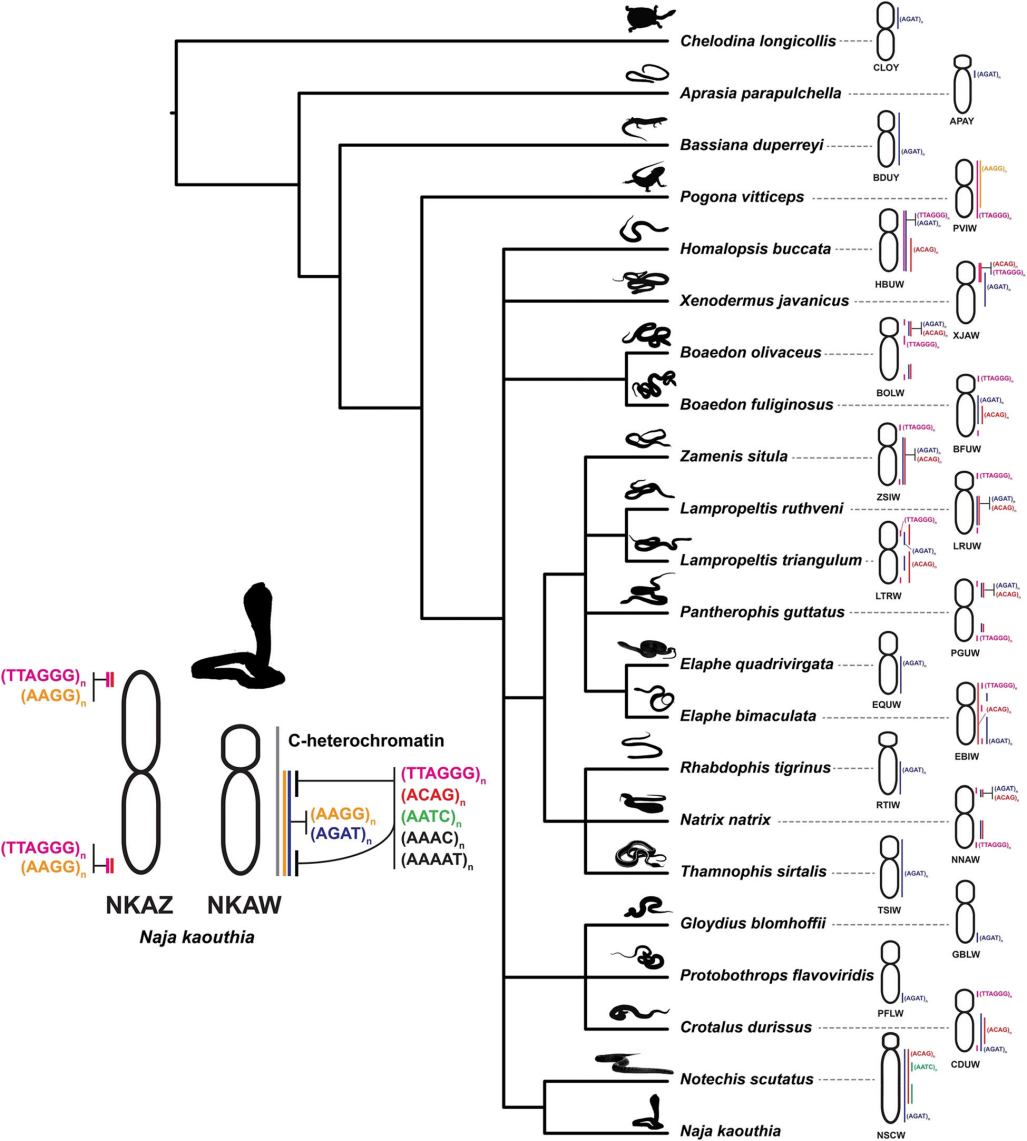
Schematic representation of microsatellite repeat motif amplification on the Siamese cobra W chromosome and other amniote sex chromosomes sharing the same motifs. Phylogeny was partially derived from Pyron et al. [43]. Regions where the microsatellite repeat motifs were hybridized are shown to the right of the chromosomes. Chromosome numbers indicate the pink-tailed worm-lizard (*Aprasia parapulchella*, APA), the Japanese striped snake (*Elaphe quadrivirgata*, EQU), the tiger keelback snake (*Rhabdophis tigrinus*, RTI), the habu *Protobothrops flavoviridis*, (PFL), the Japanese moccasin *Gloydius blomhoffii*, (GBL), the eastern three-lined skink (*Bassiana duperreyi*, BDU), the central bearded dragon (*Pogona vitticeps*, PVI), the eastern long-necked turtle (*Chelodina longicollis*, CLO), the tiger snake (*Notechis scutatus*, NSC), the tiger keelback snake (*Rhabdophis tigrinus*, RTI), the dragon snake (*Xenodermus javanicus*, XJA), the South American rattlesnake (*Crotalus durissus*, CDU), the masked water snake (*Homalopsis buccata*, HBU), the African house snake (*Boaedon fuliginosus*, BFU and *Boaedon olivaceus*, BOL), the milk snake (*Lampropeltis triangulum*, LTR), the Ruthven's kingsnake (*Lampropeltis ruthveni*, LRU), the corn snake (*Pantherophis guttatus*, PGU), the twin-spotted ratsnake (*Elaphe bimaculata*), the leopard snake (*Zamenis situla*, ZSI), the grass snake (*Natrix natrix*, NNA), and the garter snake (*Thamnophis sirtalis*, TSI) showing shared microsatellite repeat motifs with the Siamese cobra W chromosome. Chromosomal locations of repeat motifs in the amniotes were obtained from the following sources: *A. parapulchella* from Matsubara et al. [53], *E. quadrivirgata*, *R. tigrinus*, *P. flavoviridis* and *G. blomhoffii* from Matsubara et al. [27, 28], *B. duperreyi*, *P. vitticeps*, *C. longicollis*, *N. scutatus* and *R. tigrinus* from Matsubara et al. [26, 27], *X. javanicus* from Rovatsos et al. [54], *C. durissus*, *H. buccata*, *B. fuliginosus*, *B. olivaceus*, *L. triangulum*, *L. ruthveni*, *P. guttatus*, *E. bimaculata*, *Z. situla* and *N. natrix* from Augstenová et al. [25], and *T. sirtalis* from Perry et al. [42]

The W chromosome was smaller than the Z chromosome in the Siamese cobra, while C-banding revealed almost the entire heterochromatic nature of the W chromosome. This suggests that size reduction and heterochromatinization are correlated with W chromosome differentiation in the Siamese cobra. Our FISH analyses revealed that six microsatellite repeat motifs ((AAGG)_8_, (AGAT)_8_, (AAAC)_8_, (ACAG)_8_, (AATC)_8_, and (AAAAT)_6_) were amplified on NKAWq. Hybridization signals of six microsatellite repeat motifs also overlapped with telomeric (TTAGGG)_n_ repeats identified on the NKAWq. These results collectively suggest that the NKAW are structural complexes containing various repeat sequences on the female-specific region. A different hybridization pattern among repeat motifs indicates that each repeat type is distinct. Such amplification of microsatellite repeats and telomeric repeats in the heterochromatic region on W chromosomes has been reported in several caenophidian snakes and other squamate reptiles [25–27, 41] (Fig. 5). One microsatellite repeat motif amplified on the W chromosome in several caenophidian snakes though not for basal lineage is the banded krait minor satellite (Bkm), consisting of microsatellite repeat motif (AGAT)_n_ or (GACA)_n_ sequences, and related to the degree of ZW differentiation [25, 26, 42, 43]. This also suggests that frequent amplification of the repeats has a structural role in heterochromatinization and promotes further sex chromosome differentiation.

Amplification of (ACAG)_n_ and (AATC)_n_ was found on the W chromosome of tiger snakes (*N*. *scutatus*), while (AGAT)_n_ was also observed in the Japanese striped snake (*E. quadrivirgata*), and the tiger keelback snake (*R. tigrinus*) [26] (Fig. 5). However, the sets of microsatellite repeat motifs used in this study generally differed from those in previous reports. Therefore, it cannot be ruled out that other microsatellite repeat motifs might be amplified in the Siamese cobra. Interestingly, both (AAGG)_n_ and telomeric repeats identified on the central bearded dragon (*P. vitticeps*) W chromosome were also identified on NKAW, even though the species are only distantly related ([26, 41, 44]; Srikulnath et al. unpublished data). Similar results were also found in other amniotes (Fig. 5). This suggests that amplification of microsatellite repeat motifs occurred independently in each lineage and might represent convergent sex chromosomal differentiation among amniote sex chromosomes. It should be considered that although it is known that sex chromosomes share no homology among amniotes, evidence of synteny from several amniotes showed some overlap of partial synteny likely to have been part of an ancestral super-sex chromosome [4]. An example of such non-homologous W chromosomes of the chicken and snake sharing several repeat groups has been found in the common tiger snake (*N. scutatus*) [30]. Is it possible that amplified microsatellite repeat motifs were retained in the sex chromosomes of a common ancestor and subsequent reshuffling provided the appearance of sex chromosomes in each lineage?

### Is squamate reptile chromosome 2 part of an ancestral super-sex chromosome in amniotes?

**Fig. 6:**
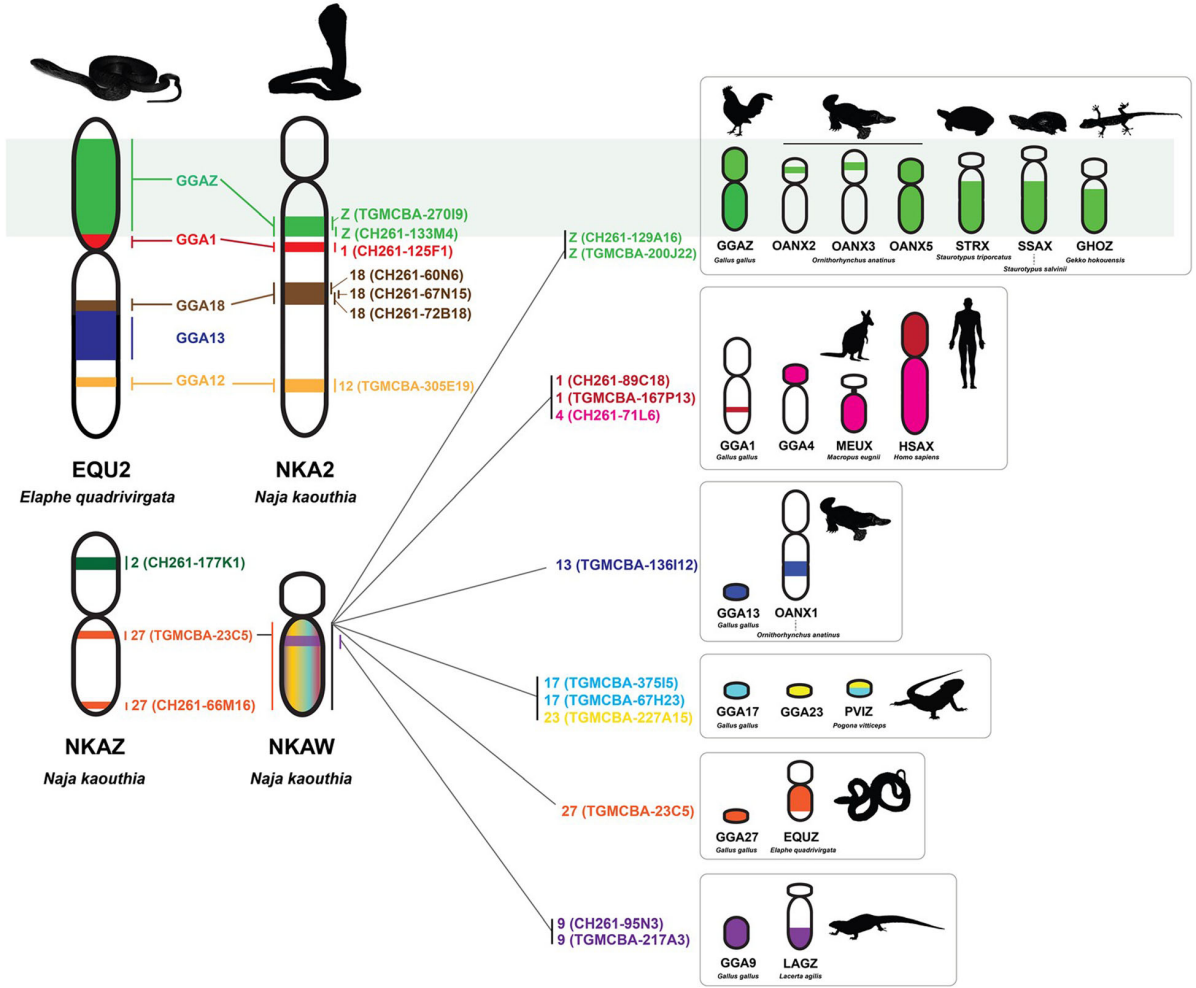
Chromosome maps of the Siamese cobra chromosome 2, and Z and W chromosomes showing homologies with the chicken, zebra finch, and several other amniotes. This map was constructed using 43 BACs derived from the chicken and zebra finch. Locations of the BACs on Siamese cobra chromosomes are shown to the right of the chromosomes. Chromosome numbers indicate the chicken (*Gallus gallus*, GGA), the green anole (*Anolis carolinensis*, ACA), the bearded dragon lizard (*Pogona vitticeps*, PVI), the Japanese four-striped rat snake (*Elaphe quadrivirgata*, EQU), humans (*Homo sapiens*, HSA), the tammar wallaby (*Macropus eugnii*, MEU), the duck-billed platypus (*Ornithorhynchus anatinus*, OAN), the giant musk turtle (*Staurotypus salvinii*, SSA), the mexican musk turtle (*Staurotypus triporcatus*, STR), the Hokou gecko (*Gekko hokouenesis*, GHO), and the sand lizard (*Lacerta agilis*, LAG) showing partial homologies with the Siamese cobra chromosomes. Partial syntenies are shown using the same color. Chromosomal locations of genes in the amniotes were obtained from the following sources: *G. gallus* from Matsuda et al. [33], *A. carolinensis* from Alföldi et al. [12], *E. quadrivirgata* from Matsubara et al. [21], *G. hokouenesis* from Kawai et al. [10], *P. vitticeps* from Ezaz et al. [13], *H. sapiens* and *M. eugnii* from Grützner et al. [55], *O. anatinus* from Veyrunes et al. [15], *S. salvinii* from Kawagoshi et al. [5] *S. triporcatus* from Montiel et al. [56], and *L. agilis* from Srikulnath et al. [14]

Except for gecko lizards and lacertid lizards, synteny on the snake Z chromosome and chromosome 2 are remarkably well conserved in most squamate reptiles [11–14, 16–18, 30]. Comparison of the chromosome maps of the Siamese cobra and other squamate reptiles revealed that subtelocentric NKA2 was homologous with submetacentric chromosome 2 of most squamate reptiles [11, 14, 16–19, 21]. This suggests that NKA2 resulted from intrachromosomal rearrangements (such as pericentric inversion or centromere repositioning) in an ancestral bi-armed macrochromosome. However, large hybridization signals like repeat sequences of one GGAZ BAC (CH261-129A16) and one TGUZ BAC (TGMCBA-200J22) observed on the Siamese cobra W chromosome were not present in other Siamese cobra chromosomes, even though these two BACs were expected to be located on NKA2. Similar results were also found in an additional eight BACs derived from GGA1, GGA4, TGU1B, TGU13, TGU17, TGU23, TGU27, which showed partial homology with various amniote sex chromosomes (Fig. 6) [4]. This suggests that these BACs contained repeat sequences shared with NKAW. Sequence analysis using RepeatMasker and MSATCOMMANDER showed 1.95–23.26% of repeat sequences for retrotransposon and 0.58–1.66 % for microsatellites repeats, also found in BACs mapped on NKAW. Transposable elements form the majority of shared elements in NKAW, supporting the co-location of heterochromatin and putative transposable elements as a result of the unchecked activity of retrotransposons in cells [45]. These repeat sequences probably drive these regions as derived sex chromosomes [3]. In agreement with the hypothesis of an ancestral amniote super-sex chromosome, observations of hybridization patterns from chicken and zebra finch BACs mapped to NKA2 and NKAW suggest that a large ancestral super-sex chromosome has strong correlation between squamate chromosome 2 (NKA2) and snake sex chromosomes. Alternatively, the shared partial synteny of amniote sex chromosomes could follow a stochastic pattern within a restricted set of species. It remains possible that the partial synteny of squamate chromosome 2 to several amniote sex chromosomes represents random homologies, given that only small sets of genes in a restricted set of species are involved. However, we also performed chromosome mapping using chicken and zebra finch BACs derived from other chromosomes and did not observe hybridization signals on NKA2, NKAZ, or NKAW. The same BACs were also used to perform chromosome mapping in other amniotes, which did not show hybridization signals such as repeat sequences ([36–38]; Singchat et al. unpublished data). Hence, mapping data on NKAW with several chicken and zebra finch BACs might not be random. However, limitations on the resolution of the FISH technique may indicate repeat sequences on NKAW, with repeats of other locations also escaping detection. At this stage, theories of independent rearrangements involving chromosomes 2, Z, and W in the snake lineage, and independent amplification of the same repeat sequences on snake and amniote sex chromosomes require further analysis of data from other squamate reptiles using FISH mapping and whole genome sequencing in order to investigate this hypothesis.

Sex-determining genes are well established in several amniotes, and orthologs or paralogs of the functional genes appear repeatedly in distantly related amniotes. This suggests the possibility of cooperative favorable linked sex chromosomes with hitchhiking effects (genes physically linked to one of the chosen sex-determining genes undergoing selective sweep) or interaction between genes [4]. The sequence of chicken and zebra finch BACs mapped to NKAW contained at least five known protein-coding genes involving sex determination and sex development pathways (Table 4). Two genes (*GPR149* and *DHX36*) located on TGU9 BAC (TGMCBA-217A3) were mapped to NKAW as a twin dot hybridization signal. This hybridization pattern is unlikely to be caused by repeat sequences. However, we have no direct evidence that these genes are involved in snake sex determination or sex development. It is likely that convergent evolution of sex chromosomes across distantly related taxa led to genomic elements that are particularly adept in a sex-determining role. Are these genes coincidental, or are there sequences in these regions that have a selectable function in sex determination? More information regarding genomic analysis and transcriptomic activity from squamate reptiles is required to test this hypothesis.

## Conclusions

This study suggests the fundamental basis on which the Siamese cobra chromosome 2 and the W sex chromosome share partial synteny and some characteristics of sex chromosomes observed in other amniotes, for example the amplification of repeat sequences. This in turn supports the hypothesis of ancestral super-sex chromosomes with overlaps of partial syntenies among amniote sex chromosomes. This might result from random distribution of microsatellite repeat motifs in each lineage as an alternative hypothesis, representing convergent sex chromosomal differentiation among amniote sex chromosomes. Further research is required to compare additional maps and sequences to explore syntenies in several squamate reptiles, using recent advances in comparative gene mapping and whole-genome sequencing. This may provide a more comprehensive understanding of the complexity of evolutionary sex determination and sex chromosomes in amniotes.

## Methods

### Specimen Collection

Two male and two female Siamese cobras (*N. kaouthia*) were collected from Snake Farm, Queen Saovabha Memorial Institute (QSMI, Bangkok). The sex of each individual was identified morphologically and confirmed using a molecular sexing approach [22–24]. Blood samples were collected from the ventral tail vein using a 23-gauge needle attached to 2-ml disposable syringes. These contained 10 mM ethylenediaminetetraacetic acid (EDTA) for DNA extraction or 75 USP unit/ml heparin for cell culture. Whole genomic DNA was extracted following the standard salting-out protocol as described previously by Supikamolseni et al. [46] and used as templates for molecular sexing. DNA quality and concentration were determined using 1% agarose gel electrophoresis and spectrophotometric analysis.

### Lymphocyte Cell culture and Chromosome Preparation

Lymphocytes from two male and two female Siamese cobras were isolated from peripheral blood, and then cultured for 5 days in RPMI 1640 medium supplemented with 15% fetal bovine serum (FBS), 3 μg/ml concanavalin A (type IV-S) (Sigma-Aldrich, St. Louis, MO, USA), 10 μg/ml lipopolysaccharide (Sigma-Aldrich), 1% phytohaemagglutinin (HA15) (Remel, Lenexa, KS, USA) and 1% Antibiotic-Antimycotic (Life Technologies-Gibco, Carlsbad, CA, USA). After 5 days lymphocytes were subject to colcemid treatment (100 ng/ml) for 60 min and fixed (3:1 methanol/acetic acid) after hypotonic treatment in 0.075 M KCl, before being harvested. The cell suspension was dropped onto clean glass slides and air-dried. The slides were kept at -80 °C until required for use.

### Karyotyping and C-banding

Morphology and size of macrochromosomes were characterized according to Turpin and Lejeune [47] and Levan et al. [48]. To examine the chromosomal distribution of constitutive heterochromatin, C-banding was performed using the standard barium hydroxide/saline/Giemsa method [49] with slight modification as follows: chromosome slides were treated with 0.2 N HCl at room temperature for 60 min and then with 5% Ba (OH)_2_ at 50 °C for 30 s, followed by 2× SSC at 65 °C for 60 min.

### Fluorescence *in situ* Hybridization (FISH) Mapping of Telomeric Repeat and Microsatellite Repeat Motifs

The chromosomal locations of telomeric (TTAGGG)_n_ sequences and 19 microsatellite repeat motifs: (CA)_15_, (GC)_15_, (GA)_15_, (AT)_15_, (CAA)_10_, (CAG)_10_, (CAT)_10_, (CGG)_10_, (GAG)_10_, (AAT)_10_, (AAGG)_8_, (AATC)_8_, (AGAT)_8_, (ACGC)_8_, (AAAT)_8_, (AAAC)_8_, (AATG)_8_, (AAATC)_6_, and (AAAAT)_6_ were determined using FISH as described previously [16, 50]. We used commercially biotin-labeled 42-bp oligonucleotide complementary to (TTAGGG)_n_ sequences and 19 commercially biotin-labeled oligonucleotide microsatellite repeat probes (Macrogen Co., Seoul, Korea), ethanol-precipitated with salmon sperm DNA, and *Escherichia coli* tRNA. After the hybridization of biotin-labeled probes to the Siamese cobra chromosomes, the probes were detected by incubating the chromosome slides with avidin labeled with fluorescein isothiocyanate (avidin-FITC; Invitrogen, CA, USA). Slides were subsequently stained with 1 μg/ml DAPI (4′, 6′-diamidino-2-phenylindole). Fluorescence hybridization signals were captured using a cooled Charge-Coupled Device (CCD) camera mounted on a ZEISS Axioplan2 microscope and processed using MetaSystems ISIS v.5.2.8 software (MetaSystems, Altlussheim, Germany).

### Isolation, Amplification and Labeling of Chicken and Zebra finch Bacterial Artificial Chromosomes (BACs)

Chicken (*Gallus gallus*) and zebra finch (*Taeniopygia guttata*) BACs were applied for cross-species FISH mapping according to a range of the proportion of conserved elements shared across multiple species. Due to the high degree of apparent genome conservation between avian and reptilian species ([36–38]; Singchat et al. unpublished data), these sets of BACs were applied to the Siamese cobras. All BACs were anchored to the chicken and zebra finch genome assembly by linkage and sequencing. Twenty-six chicken and seventeen zebra finch BACs, comprising chicken chromosome 1 (GGA1), GGA2p, GGA4, GGA5, GGA9, GGA15, GGA17, GGA18, GGA23, GGA27 and GGAZ, and zebra finch chromosome 1 (TGU1B), TGU4A, TGU5, TGU9, TGU12, TGU13, TGU15, TGU17, TGU23, TGU27 and TGUZ, were isolated using the Qiagen Miniprep Kit (Qiagen, Manchester, UK) prior to amplification and direct labeling by nick translation (Roche, Welwyn Garden City, UK). Probes were labeled with Texas Red 12-dUTP (Invitrogen Corporation and Applied Biosystems Inc., Carlsbad, California, US) and FITC-12-UTP (Roche) prior to purification using the Qiagen Nucleotide Removal Kit (Qiagen).

### Cross-Species Bacterial Artificial Chromosome (BAC) Fluorescence *in situ* Hybridization (FISH) Mapping

Chromosome slides were dehydrated through an ethanol series (2 min each in 2×SSC, 70%, 85%, and 100% ethanol at room temperature). Probes were diluted in a Hybridization Solution I (Cytocell Ltd., Cambridge, UK) with Chicken Hybloc (Insight Biotechnology limited, Wembley, London, UK) and applied to the Siamese cobra chromosomes on a 37 °C hotplate before being sealed with rubber cement. Probe and target DNA were simultaneously denatured on a 75 °C hotplate for 2 min prior to hybridization in a humidified chamber at 37 °C for 72 h. Slides were washed post-hybridization for 30 s in 2 × SSC/0.05% Tween 20 at room temperature and then counterstained using VECTASHIELD antifade mounting medium with DAPI (Vector Laboratories, Inc., Burlingame, CA, USA). Images were captured using an Olympus BX61 epifluorescence microscope with a cooled CCD camera and SmartCapture system (Digital Scientific UK Ltd., Cambridge, UK). Confirmation of BAC signal order was achieved by dual color experiments where Texas Red 12-dUTP- and FITC-12-UTP- labeled probes were hybridized simultaneously.

### Candidate Bacterial Artificial Chromosome (BAC) Sequence Analysis

Gene position and gene structure in each candidate BAC were derived from ENSEMBL (ensembl.org/Gallus_gallus/Info/Annotation). The CpG content and repeat information were retrieved from the UCSC Genome Browser http://genome.ucsc.edu/ for the corresponding gene build. Simultaneously, each BAC sequence was subjected to homology search using Blastx and Blastn (RefSeq *G. gallus* and *T. guttata*). Repeat data were identified using Arian Smit's Repeat Masker program [51], which screens DNA sequences for interspersed repeats and low complexity DNA sequences. Query species were assumed to be Vertebrata, Metazoa, Repeat Masker Combined Database; Dfam_Consensus-20170127 and RepBase-20170127, run with rmblastn version 2.2.27+ [51]. Microsatellite repeat motifs were scanned from candidate BAC sequences using MSATCOMMANDER [52]. Parameters of the minimum number of repetitions were set at 10, 6, 4, 4, 4, and 4 for mono, di, tri, tetra, penta, and hexanucleotide motifs, respectively.

## Additional files

Additional file 1: **Figure S1.** Chromosomal locations of microsatellite repeat motifs in the Siamese cobra (*Naja kaouthia*). Hybridization patterns of FITC-labeled (CA)_15_ (**a**), (AT)_15_ (**b**), (GC)_15_ (**c**), (CAT)_10_ (**d**), and (AAT)_10_ (**e**) on DAPI-stained chromosomes. Scale bar represents 10 μm. (JPG 175 kb)

Additional file 2: **Figure S2.** Cytogenetic map of the Siamese cobra (*Naja kaouthia*), which shows chromosome homologies with chicken and zebra finch. This map was constructed with 25 chicken and zebra finch BACs mapped on the Siamese cobra macrochromosomes. Locations of BACs are shown to the right of the Siamese cobra chromosomes. The chromosome numbers show the chromosomes of the chicken (*Gallus gallus*, GGA) and zebra finch (*Taeniopygia guttata*, TGU), which show homologies with the Siamese cobra chromosomes. (JPG 118 kb)

Additional file 3: **Table S1.** Comparison of major classes of repeat sequences in chicken and zebra finch BACs mapped on the Siamese cobra Z chromosome. (DOCX 14 kb)

Additional file 4: **Table S2.** Comparison of frequencies of microsatellite repeat motifs in chicken and zebra finch BACs mapped on the Siamese cobra Z chromosome. (DOCX 13 kb)

Additional file 5: **Table S3.** Comparison of major classes of repeat sequences in chicken and zebra finch BACs mapped on the Siamese cobra chromosome 2. (DOCX 15 kb)

Additional file 6: **Table S4.** Comparison of frequencies of microsatellite repeat motifs in chicken and zebra finch BACs mapped on the Siamese cobra chromosome 2. (DOCX 14 kb)

Additional file 7: **Table S5.** Comparison of major classes of repeat sequences in 22 chicken and zebra finch BACs mapped on the Siamese cobra chromosome 1 and microchromosomes. (DOCX 23 kb)

Additional file 8: **Table S6.** Comparison of frequencies of microsatellite repeat motifs in 22 chicken and zebra finch BACs mapped on the Siamese cobra chromosome 1 and microchromosomes. (DOCX 19 kb)

## Abbreviations

ACA: *Anolis carolinensis*
APA: *Aprasia parapulchella*
BACs: bacterial artificial chromosomes
BDU: *Bassiana duperreyi*
BFU: *Boaedon fuliginosus*
Bkm: banded krait minor satellite
BOL: *Boaedon olivaceus*
CCD: Charge-Coupled Device
CDU: *Crotalus durissus*
CLO: *Chelodina longicollis*
DAPI: 4′, 6-diamidino-2-phenylindole
EBI: *Elaphe bimaculata*
EQU: *Elaphe quadrivirgata*
FBS: fetal bovine serum
FISH: Fluorescent in situ hybridization
FITC: Fluorescein isothiocyanate
GBL: *Gloydius blomhoffii*
GGA: *Gallus gallus*
GHO: *Gekko hokouenesis*
HBU: *Homalopsis buccata*
HSA: *Homo sapiens*
LAG: *Lacerta agilis*
LRU: *Lampropeltis ruthveni*
LTR: *Lampropeltis triangulum*
MEU: *Macropus eugnii*
NKA: *Naja kaouthia*
NNA: *Natrix natrix*
NSC: *Notechis scutatus*
OAN: *Ornithorhynchus anatinus*
PFL: *Protobothrops flavoviridis*
PGU: *Pantherophis guttatus*
PVI: *Pogona vitticeps*
QSMI: Queen Saovabha Memorial Institute
RTI: *Rhabdophis tigrinus*
SSA: *Staurotypus salvinii*
STR: *Staurotypus triporcatus*
TGU: *Taeniopygia guttata*
TSI: *Thamnophis sirtalis*
XJA: *Xenodermus javanicus*
ZSI: *Zamenis situla*
