# Supplementary figures and images for: Chromosome map of the Siamese cobra: did partial synteny of sex chromosomes in the amniote represent “a hypothetical ancestral super-sex chromosome” or random distribution?
Source: BMC Genomics. 2018 Dec 17;19:939. doi: 10.1186/s12864-018-5293-6 (PMC6296137; doi:10.1186/s12864-018-5293-6)

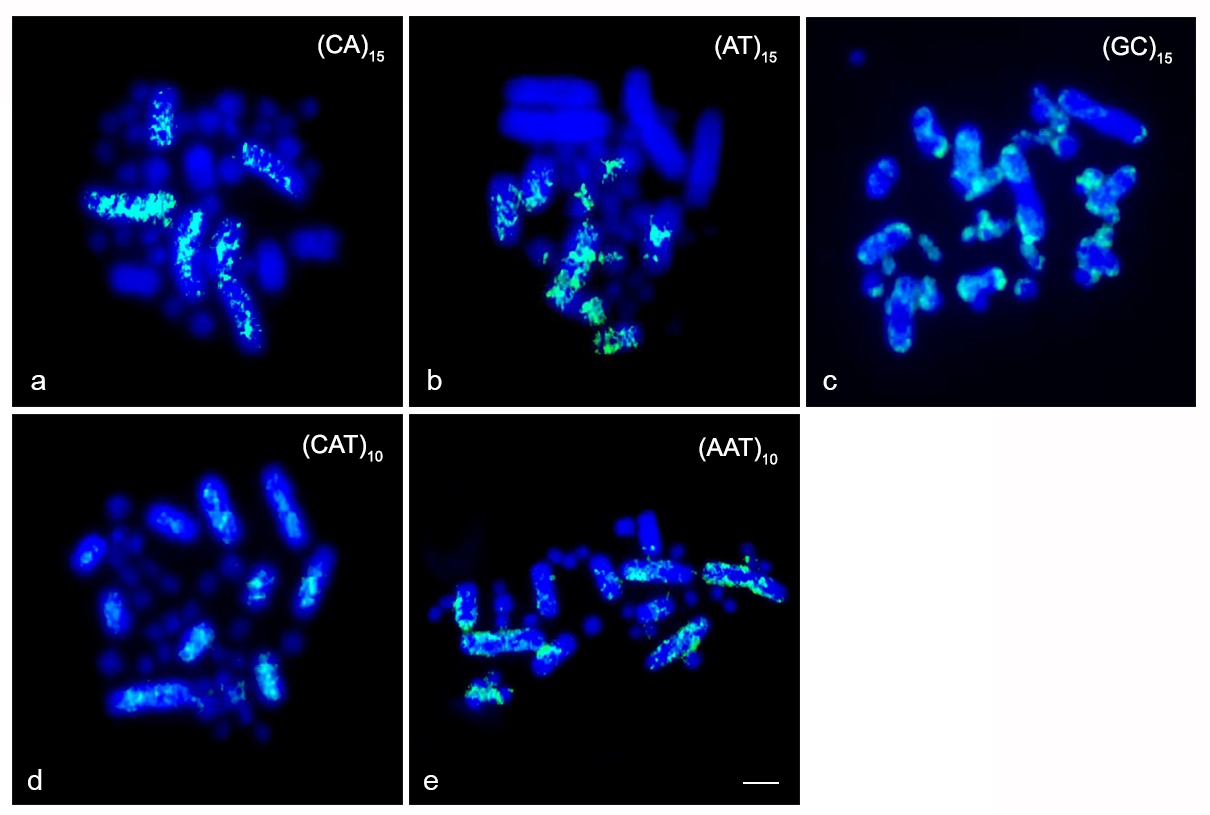

Supplement: Supplementary file 1 — Figure S1. Chromosomal locations of microsatellite repeat motifs in the Siamese cobra (Naja kaouthia). Hybridization patterns of FITC-labeled (CA)15 (a), (AT)15 (b), (GC)15 (c), (CAT)10 (d), and (AAT)10 (e) on DAPI-stained chromosomes. Scale bar represents 10 μm. (JPG 175 kb) [file 12864_2018_5293_MOESM1_ESM.jpg]

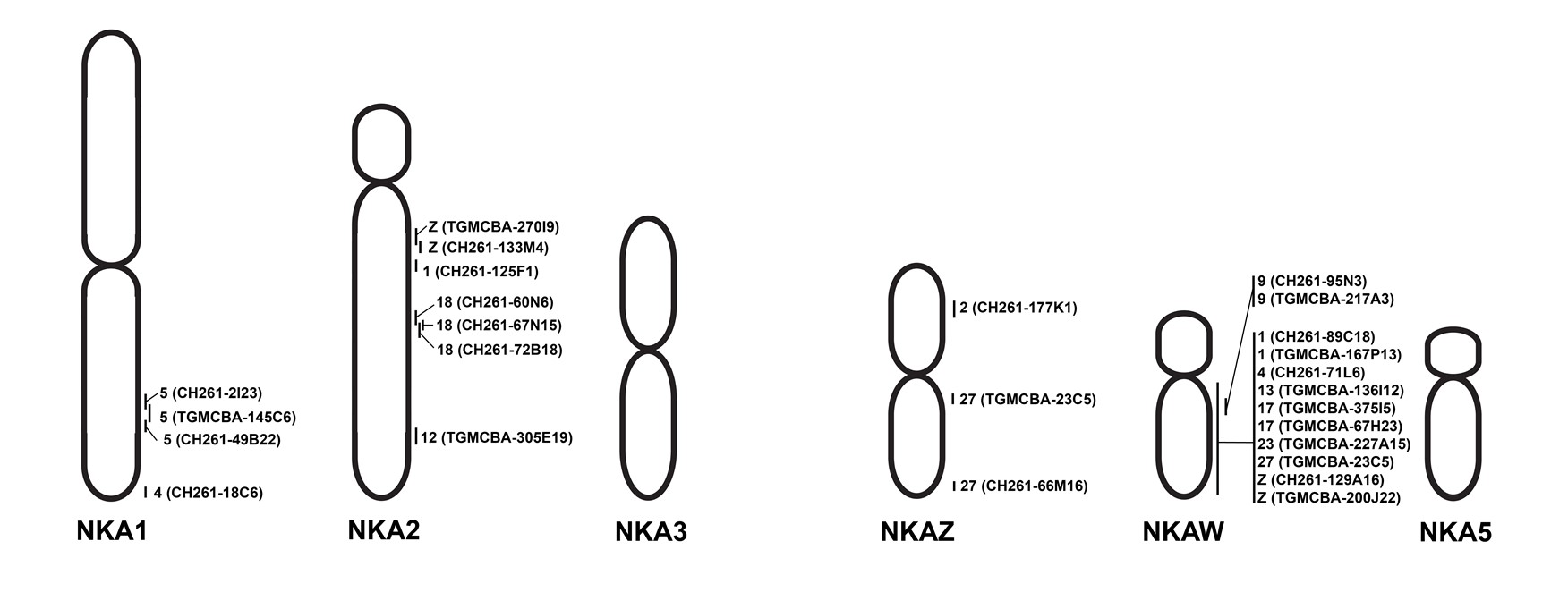

Supplement: Supplementary file 2 — Figure S2. Cytogenetic map of the Siamese cobra (Naja kaouthia), which shows chromosome homologies with chicken and zebra finch. This map was constructed with 25 chicken and zebra finch BACs mapped on the Siamese cobra macrochromosomes. Locations of BACs are shown to the right of the Siamese cobra chromosomes. The chromosome numbers show the chromosomes of the chicken (Gallus gallus, GGA) and zebra finch (Taeniopygia guttata, TGU), which show homologies with the Siamese cobra chromosomes. (JPG 118 kb) [file 12864_2018_5293_MOESM2_ESM.jpg]
