# Supplementary material for: Chromosome map of the Siamese cobra: did partial synteny of sex chromosomes in the amniote represent “a hypothetical ancestral super-sex chromosome” or random distribution?
Source: BMC Genomics. 2018 Dec 17;19:939. doi: 10.1186/s12864-018-5293-6 (PMC6296137; doi:10.1186/s12864-018-5293-6)
Supplement: Supplementary file 3 — Table S1. Comparison of major classes of repeat sequences in chicken and zebra finch BACs mapped on the Siamese cobra Z chromosome. (DOCX 14 kb) [file 12864_2018_5293_MOESM3_ESM.docx]

**Table S1** Comparison of major classes of repeat sequences in chicken and zebra finch BACs mapped on the Siamese cobra Z chromosome.

|  | **2p CH261-177K1** | | | **CH261-66M16** | | | **TGMCBA-23C5** | | |
| --- | --- | --- | --- | --- | --- | --- | --- | --- | --- |
|  | NE | LO | % | NE | LO | % | NE | LO | % |
| %GC |  |  | 41.07 |  |  | 51.29 |  |  | 48.70 |
| Satellites | 0 | 0 | 0 | 0 | 0 | 0 | 0 | 0 | 0 |
| Simple repeats | 43 | 1388 | 0.57 | 34 | 1581 | 0.89 | 40 | 2247 | 1.11 |
|  |  |  |  |  |  |  |  |  |  |
| Retroelement | 35 | 9359 | 3.85 | 9 | 920 | 0.52 | 161 | 47194 | 23.26 |
| 1) SINEs | 5 | 639 | 0.26 | 0 | 0 | 0 | 2 | 139 | 0.07 |
| 2) LINEs | 27 | 8537 | 3.52 | 7 | 801 | 0.45 | 117 | 25323 | 12.48 |
| 3) LTR elements | 3 | 183 | 0.08 | 2 | 119 | 0.07 | 42 | 21732 | 10.71 |
|  |  |  |  |  |  |  |  |  |  |
| DNA transposons | 20 | 2730 | 1.12 | 1 | 57 | 0.03 | 6 | 979 | 0.48 |
|  |  |  |  |  |  |  |  |  |  |
| Unclassified | 1 | 160 | 0.07 | 0 | 0 | 0 | 0 | 0 | 0 |
|  |  |  |  |  |  |  |  |  |  |
| Total interspersed repeats |  | 12249 | 5.05 |  | 977 | 0.55 |  | 48173 | 23.74 |

NE; number of elements

LO; length occupied

%; sequence percentage
