# Supplementary material for: Chromosome map of the Siamese cobra: did partial synteny of sex chromosomes in the amniote represent “a hypothetical ancestral super-sex chromosome” or random distribution?
Source: BMC Genomics. 2018 Dec 17;19:939. doi: 10.1186/s12864-018-5293-6 (PMC6296137; doi:10.1186/s12864-018-5293-6)
Supplement: Supplementary file 4 — Table S2. Comparison of frequencies of microsatellite repeat motifs in chicken and zebra finch BACs mapped on the Siamese cobra Z chromosome. (DOCX 13 kb) [file 12864_2018_5293_MOESM4_ESM.docx]

**Table S2** Comparison of frequencies of microsatellite repeat motifs in chicken and zebra finch BACs mapped on the Siamese cobra Z chromosome.

| **Chicken chromosome** | **BAC** | **Size (bp)** | **Number of repeats** | **Top five repeat motifs** | | | | | | | | | | | | | | |
| --- | --- | --- | --- | --- | --- | --- | --- | --- | --- | --- | --- | --- | --- | --- | --- | --- | --- | --- |
|  |  |  |  | **type** | **bp** | **%** | **type** | **bp** | **%** | **type** | **bp** | **%** | **type** | **bp** | **%** | **type** | **bp** | **%** |
| 2p | 2p CH261-177K1 | 242,791 | 21 | (AT)_37_ | 74 | 0.0305 | (ATCCC)_8_ | 40 | 0.0165 | (GT)_20_ | 40 | 0.0165 | (AAT)_8_ | 24 | 0.0099 | (ATT)_7_ | 21 | 0.0086 |
| 27 | CH261-66M16 | 177,166 | 18 | (CCT)_25_ | 75 | 0.0423 | (ATT)_14_ | 42 | 0.0237 | (AC)_15_ | 30 | 0.0169 | (CGG)_9_ | 27 | 0.0152 | (ACC)_9_ | 27 | 0.0152 |
| 27 | TGMCBA-23C5 | 202,884 | 33 | (GGAT)_43_ | 172 | 0.0848 | (AAACCT)_11_ | 66 | 0.0325 | (CCCTT)_9_ | 45 | 0.0222 | (ATCC)_10_ | 40 | 0.0197 | (AGC)_12_ | 36 | 0.0177 |
