# Supplementary material for: Chromosome map of the Siamese cobra: did partial synteny of sex chromosomes in the amniote represent “a hypothetical ancestral super-sex chromosome” or random distribution?
Source: BMC Genomics. 2018 Dec 17;19:939. doi: 10.1186/s12864-018-5293-6 (PMC6296137; doi:10.1186/s12864-018-5293-6)
Supplement: Supplementary file 5 — Table S3. Comparison of major classes of repeat sequences in chicken and zebra finch BACs mapped on the Siamese cobra chromosome 2. (DOCX 15 kb) [file 12864_2018_5293_MOESM5_ESM.docx]

**Table S3** Comparison of major classes of repeat sequences in chicken and zebra finch BACs mapped on the Siamese cobra chromosome 2.

|  | **CH261-125F1** | | | **TGMCBA-305E19** | | | **CH261-60N6** | | | **CH261-67N15** | | | **CH261-72B18** | | | **CH261-133M4** | | | **TGMCBA-270I9** | | |
| --- | --- | --- | --- | --- | --- | --- | --- | --- | --- | --- | --- | --- | --- | --- | --- | --- | --- | --- | --- | --- | --- |
|  | **NE** | **LO** | **%** | **NE** | **LO** | **%** | **NE** | **LO** | **%** | **NE** | **LO** | **%** | **NE** | **LO** | **%** | **NE** | **LO** | **%** | **NE** | **LO** | **%** |
| %GC |  |  | 48.49 |  |  | 45.38 |  |  | 52.23 |  |  | 43.85 |  |  | 44.63 |  |  | 40.04 |  |  | 41.79 |
| Satellites | 2 | 306 | 0.15 | 0 | 0 | 0 | 0 | 0 | 0 | 0 | 0 | 0 | 0 | 0 | 0 | 1 | 8 | 0.00 | 0 | 0 | 0.00 |
| Simple repeats | 50 | 2047 | 0.99 | 46 | 2245 | 1.34 | 58 | 2612 | 1.12 | 31 | 1097 | 0.6 | 27 | 877 | 0.51 | 87 | 5224 | 2.72 | 62 | 3132 | 2.14 |
|  |  |  |  |  |  |  |  |  |  |  |  |  |  |  |  |  |  |  |  |  |  |
| Retroelement | 55 | 21483 | 10.36 | 41 | 12904 | 7.69 | 14 | 2784 | 1.20 | 68 | 32793 | 17.82 | 15 | 5867 | 3.41 | 32 | 13324 | 6.93 | 25 | 8572 | 5.86 |
| 1) SINEs | 2 | 115 | 0.06 | 1 | 62 | 0.04 | 1 | 99 | 0.04 | 0 | 0 | 0 | 0 | 0 | 0 | 3 | 711 | 0.37 | 0 | 0 | 0 |
| 2) LINEs | 48 | 20181 | 9.74 | 30 | 8277 | 4.93 | 13 | 2685 | 1.15 | 58 | 29540 | 16.05 | 11 | 5003 | 2.9 | 26 | 12000 | 6.24 | 17 | 6193 | 4.23 |
| 3) LTR elements | 5 | 1187 | 0.57 | 10 | 4565 | 2.72 | 0 | 0 | 0 | 10 | 3253 | 1.77 | 4 | 864 | 0.5 | 3 | 613 | 0.32 | 8 | 2379 | 1.63 |
|  |  |  |  |  |  |  |  |  |  |  |  |  |  |  |  |  |  |  |  |  |  |
| DNA transposons | 7 | 1401 | 0.68 | 5 | 262 | 0.16 | 2 | 135 | 0.06 | 14 | 3375 | 1.83 | 3 | 117 | 0.07 | 11 | 4203 | 2.19 | 1 | 64 | 0.04 |
|  |  |  |  |  |  |  |  |  |  |  |  |  |  |  |  |  |  |  |  |  |  |
| Unclassified | 1 | 45 | 0.02 | 1 | 33 | 0.02 | 0 | 0 | 0 | 0 | 0 | 0 | 0 | 0 | 0 | 3 | 483 | 0.25 | 0 | 0 | 0 |
|  |  |  |  |  |  |  |  |  |  |  |  |  |  |  |  |  |  |  |  |  |  |
| Total interspersed repeats |  | 22929 | 11.06 |  | 13199 | 7.86 |  | 2919 | 1.25 |  | 36168 | 19.65 |  | 5984 | 3.47 |  | 18010 | 9.37 |  | 8636 | 5.90 |
