# Supplementary material for: Chromosome map of the Siamese cobra: did partial synteny of sex chromosomes in the amniote represent “a hypothetical ancestral super-sex chromosome” or random distribution?
Source: BMC Genomics. 2018 Dec 17;19:939. doi: 10.1186/s12864-018-5293-6 (PMC6296137; doi:10.1186/s12864-018-5293-6)
Supplement: Supplementary file 6 — Table S4. Comparison of frequencies of microsatellite repeat motifs in chicken and zebra finch BACs mapped on the Siamese cobra chromosome 2. (DOCX 14 kb) [file 12864_2018_5293_MOESM6_ESM.docx]

**Table S4** Comparison of frequencies of microsatellite repeat motifs in chicken and zebra finch BACs mapped on the Siamese cobra chromosome 2.

| **Chicken chromosome** | **BAC** | **size (bp)** | **number of repeats** | **top five repeat motifs** | | | | | | | | | | | | | | |
| --- | --- | --- | --- | --- | --- | --- | --- | --- | --- | --- | --- | --- | --- | --- | --- | --- | --- | --- |
|  |  |  |  | **type** | **bp** | **%** | **type** | **bp** | **%** | **type** | **bp** | **%** | **type** | **bp** | **%** | **type** | **bp** | **%** |
| 1 | CH261-125F1 | 207,294 | 29 | (AT)_23_ | 46 | 0.0222 | (AGG)_12_ | 36 | 0.0174 | (AAAC)_8_ | 32 | 0.0154 | (AAT)_10_ | 30 | 0.0145 | (AC)_15_ | 30 | 0.0145 |
| 12 | TGMCBA-305E19 | 167,890 | 14 | (CTTTT)_14_ | 50 | 0.0298 | (AT)_22_ | 44 | 0.0262 | (ATT)_13_ | 39 | 0.0232 | (GT)_18_ | 36 | 0.0214 | (ACT)_10_ | 30 | 0.0179 |
| 18 | CH261-60N6 | 232,938 | 19 | (AGG)_29_ | 87 | 0.0373 | (GCT)_12_ | 36 | 0.0155 | (GGT)_11_ | 33 | 0.0142 | (ACGGG)_5_ | 25 | 0.0107 | (ATCC)_6_ | 24 | 0.0103 |
| 18 | CH261-67N15 | 184,029 | 7 | (AT)_20_ | 40 | 0.0217 | (CCCGG)_4_ | 20 | 0.0109 | (ATC)_4_ | 12 | 0.0065 | (GCT)_4_ | 12 | 0.0065 | (CCT)_4_ | 12 | 0.0065 |
| 18 | CH261-72B18 | 172,245 | 9 | (GT)_11_ | 22 | 0.0128 | (GCT)_6_ | 18 | 0.0105 | (CCCT)_4_ | 16 | 0.0093 | (CT)_7_ | 14 | 0.0081 | (AG)_7_ | 14 | 0.0081 |
| Z | CH261-133M4 | 192,220 | 58 | (CCCTT)_68_ | 276 | 0.1769 | (AAAG)_69_ | 340 | 0.1436 | (CCTCT)_40_ | 200 | 0.1040 | (AGAGG)_37_ | 185 | 0.0962 | (AAGG)_34_ | 136 | 0.0708 |
| Z | TGMCBA-270I9 | 146,379 | 30 | (CTCTT)_41_ | 144 | 0.1400 | (CTTTTT)_24_ | 225 | 0.0984 | (CTTTT)_24_ | 120 | 0.0820 | (ATTTTT)_19_ | 114 | 0.0779 | (AAGAG)_15_ | 75 | 0.0512 |
