# Supplementary material for: Chromosome map of the Siamese cobra: did partial synteny of sex chromosomes in the amniote represent “a hypothetical ancestral super-sex chromosome” or random distribution?
Source: BMC Genomics. 2018 Dec 17;19:939. doi: 10.1186/s12864-018-5293-6 (PMC6296137; doi:10.1186/s12864-018-5293-6)
Supplement: Supplementary file 7 — Table S5. Comparison of major classes of repeat sequences in 22 chicken and zebra finch BACs mapped on the Siamese cobra chromosome 1 and microchromosomes. (DOCX 23 kb) [file 12864_2018_5293_MOESM7_ESM.docx]

**Table S5** Comparison of major classes of repeat sequences in 22 chicken and zebra finch BACs mapped on the Siamese cobra chromosome 1 and microchromosomes.

|  | **CH261-184E5** | | | **CH261-36B5** | | | **CH261-18J16** | | | **CH261-83O13** | | | **CH261-18C6** | | | **CH261-85H10** | | |
| --- | --- | --- | --- | --- | --- | --- | --- | --- | --- | --- | --- | --- | --- | --- | --- | --- | --- | --- |
|  | **NE** | **LO** | **%** | **NE** | **LO** | **%** | **NE** | **LO** | **%** | **NE** | **LO** | **%** | **NE** | **LO** | **%** | **NE** | **LO** | **%** |
| %GC |  |  | 38.14 |  |  | 39.33 |  |  | 41.02 |  |  | 42.86 |  |  | 37.46 |  |  | 42.57 |
| Satellites | 0 | 0 | 0 | 0 | 0 | 0 | 1 | 66 | 0.03 | 1 | 103 | 0.04 | 0 | 0 | 0 | 0 | 0 | 0 |
| Simple repeats | 67 | 2673 | 1.14 | 76 | 2864 | 1.38 | 62 | 2186 | 0.85 | 76 | 4246 | 1.77 | 61 | 2043 | 0.81 | 66 | 2467 | 1.01 |
|  |  |  |  |  |  |  |  |  |  |  |  |  |  |  |  |  |  |  |
| Retroelement | 43 | 12465 | 5.33 | 19 | 4006 | 1.93 | 73 | 19368 | 7.50 | 64 | 20876 | 8.71 | 46 | 13523 | 5.35 | 54 | 11102 | 4.53 |
| 1) SINEs | 2 | 61 | 0.03 | 4 | 543 | 0.26 | 4 | 478 | 0.19 | 0 | 0 | 0 | 2 | 289 | 0.11 | 1 | 47 | 0.02 |
| 2) LINEs | 40 | 11870 | 5.07 | 14 | 3186 | 1.53 | 65 | 18133 | 7.02 | 60 | 19359 | 8.08 | 33 | 9639 | 3.81 | 45 | 10364 | 4.23 |
| 3) LTR elements | 1 | 534 | 0.23 | 1 | 277 | 0.13 | 4 | 757 | 0.29 | 4 | 1517 | 0.63 | 11 | 3595 | 1.42 | 8 | 691 | 0.28 |
|  |  |  |  |  |  |  |  |  |  |  |  |  |  |  |  |  |  |  |
| DNA transposons | 10 | 2430 | 1.04 | 14 | 5037 | 2.43 | 20 | 4342 | 1.68 | 17 | 2808 | 1.17 | 15 | 5077 | 2.01 | 10 | 1900 | 0.78 |
|  |  |  |  |  |  |  |  |  |  |  |  |  |  |  |  |  |  |  |
| Unclassified | 0 | 0 | 0 | 0 | 0 | 0 | 1 | 98 | 0.04 | 3 | 512 | 0.21 | 0 | 0 | 0 | 1 | 31 | 0.01 |
|  |  |  |  |  |  |  |  |  |  |  |  |  |  |  |  |  |  |  |
| Total interspersed repeats |  | 14895 | 6.37 |  | 9043 | 4.36 |  | 23808 | 9.22 |  | 24196 | 10.09 |  | 18600 | 7.36 |  | 18010 | 9.37 |

|  | **TGMCBA-200G5** | | | **TGMCBA-280M7** | | | **TGMCBA-330J11** | | | **CH261-2I23** | | | **TGMCBA-145C6** | | | **CH261-49B22** | | |
| --- | --- | --- | --- | --- | --- | --- | --- | --- | --- | --- | --- | --- | --- | --- | --- | --- | --- | --- |
|  | **NE** | **LO** | **%** | **NE** | **LO** | **%** | **NE** | **LO** | **%** | **NE** | **LO** | **%** | **NE** | **LO** | **%** | **NE** | **LO** | **%** |
| %GC |  |  | 41.80 |  |  | 38.72 |  |  | 45.80 |  |  | 41.84 |  |  | 40.43 |  |  | 43.01 |
| Satellites | 0 | 0 | 0 | 0 | 0 | 0 | 0 | 0 | 0 | 0 | 0 | 0 | 0 | 0 | 0 | 0 | 0 | 0 |
| Simple repeats | 25 | 1072 | 0.79 | 27 | 1038 | 0.78 | 9 | 310 | 0.21 | 55 | 1817 | 1.08 | 42 | 1400 | 0.87 | 73 | 3291 | 1.70 |
|  |  |  |  |  |  |  |  |  |  |  |  |  |  |  |  |  |  |  |
| Retroelement | 11 | 2381 | 1.76 | 22 | 4963 | 3.74 | 9 | 2222 | 1.51 | 21 | 4526 | 2.70 | 25 | 12459 | 7.71 | 28 | 3258 | 1.69 |
| 1) SINEs | 0 | 0 | 0 | 2 | 204 | 0.15 | 0 | 0 | 0 | 3 | 325 | 0.19 | 2 | 355 | 0.22 | 2 | 180 | 0.09 |
| 2) LINEs | 6 | 1025 | 0.76 | 12 | 1096 | 0.83 | 6 | 1241 | 0.84 | 13 | 3344 | 1.99 | 9 | 2305 | 1.43 | 21 | 2798 | 1.45 |
| 3) LTR elements | 5 | 1356 | 1.00 | 8 | 3663 | 2.76 | 3 | 981 | 0.67 | 5 | 857 | 0.51 | 14 | 9799 | 6.07 | 5 | 280 | 0.14 |
|  |  |  |  |  |  |  |  |  |  |  |  |  |  |  |  |  |  |  |
| DNA transposons | 4 | 843 | 0.62 | 1 | 105 | 0.08 | 9 | 704 | 0.48 | 12 | 2070 | 1.23 | 8 | 1706 | 1.06 | 12 | 2025 | 1.05 |
|  |  |  |  |  |  |  |  |  |  |  |  |  |  |  |  |  |  |  |
| Unclassified | 0 | 0 | 0 | 0 | 0 | 0 | 1 | 154 | 0.10 | 3 | 243 | 0.14 | 2 | 325 | 0.20 | 3 | 471 | 0.24 |
|  |  |  |  |  |  |  |  |  |  |  |  |  |  |  |  |  |  |  |
| Total interspersed repeats |  | 3224 | 2.38 |  | 5068 | 3.82 |  | 3080 | 2.09 |  | 6839 | 4.07 |  | 14490 | 8.97 |  | 5754 | 2.98 |

|  | **CH261-183N19** | | | **CH261-187M16** | | | **CH261-90P23** | | | **TGMCBA-266G23** | | | **CH261-69M11** | | | **CH261-191G17** | | |
| --- | --- | --- | --- | --- | --- | --- | --- | --- | --- | --- | --- | --- | --- | --- | --- | --- | --- | --- |
|  | **NE** | **LO** | **%** | **NE** | **LO** | **%** | **NE** | **LO** | **%** | **NE** | **LO** | **%** | **NE** | **LO** | **%** | **NE** | **LO** | **%** |
| %GC |  |  | 42.25 |  |  | 48.84 |  |  | 47.10 |  |  | 42.58 |  |  | 47.80 |  |  | 49.17 |
| Satellites | 0 | 0 | 0 | 0 | 0 | 0 | 0 | 0 | 0 | 0 | 0 | 0 | 0 | 0 | 0 | 1 | 15 | 0.01 |
| Simple repeats | 33 | 1144 | 0.66 | 54 | 2216 | 1.17 | 42 | 1348 | 0.73 | 26 | 846 | 0.64 | 44 | 1940 | 1.15 | 63 | 2769 | 1.27 |
|  |  |  |  |  |  |  |  |  |  |  |  |  |  |  |  |  |  |  |
| Retroelement | 35 | 11962 | 6.87 | 15 | 2338 | 1.24 | 16 | 2563 | 1.38 | 19 | 3256 | 2.47 | 10 | 1999 | 1.18 | 23 | 7132 | 3.28 |
| 1) SINEs | 0 | 0 | 0 | 0 | 0 | 0 | 4 | 313 | 0.17 | 3 | 573 | 0.44 | 0 | 0 | 0 | 1 | 80 | 0.04 |
| 2) LINEs | 32 | 11536 | 6.63 | 12 | 2201 | 1.17 | 11 | 2217 | 1.20 | 16 | 2683 | 2.04 | 9 | 1959 | 1.16 | 20 | 6396 | 2.94 |
| 3) LTR elements | 3 | 426 | 0.24 | 3 | 137 | 0.07 | 1 | 33 | 0.02 | 0 | 0 | 0 | 1 | 40 | 0.02 | 2 | 656 | 0.30 |
|  |  |  |  |  |  |  |  |  |  |  |  |  |  |  |  |  |  |  |
| DNA transposons | 6 | 630 | 0.36 | 0 | 0 | 0 | 9 | 1252 | 0.68 | 4 | 917 | 0.70 | 5 | 570 | 0.34 | 4 | 786 | 0.36 |
|  |  |  |  |  |  |  |  |  |  |  |  |  |  |  |  |  |  |  |
| Unclassified | 0 | 0 | 0 | 0 | 0 | 0 | 2 | 148 | 0.08 | 0 | 0 | 0 | 1 | 58 | 0.03 | 0 | 0 | 0 |
|  |  |  |  |  |  |  |  |  |  |  |  |  |  |  |  |  |  |  |
| Total interspersed repeats |  | 12592 | 7.24 |  | 2338 | 1.24 |  | 3963 | 2.14 |  | 4227 | 3.21 |  | 2627 | 1.56 |  | 7918 | 3.64 |

|  | **CH261-105P1** | | | **CH261-49G9** | | | **TGMCBA-173N15** | | | **TGMCBA-48O8** | | |
| --- | --- | --- | --- | --- | --- | --- | --- | --- | --- | --- | --- | --- |
|  | **NE** | **LO** | **%** | **NE** | **LO** | **%** | **NE** | **LO** | **%** | **NE** | **LO** | **%** |
| %GC |  |  | 51.16 |  |  | 50.73 |  |  | 50.02 |  |  | 52.05 |
| Satellites | 0 | 0 | 0 | 2 | 143 | 0.06 | 0 | 0 | 0 | 0 | 0 | 0 |
| Simple repeats | 35 | 1433 | 0.78 | 37 | 1433 | 0.65 | 36 | 1522 | 1.02 | 48 | 2206 | 1.45 |
|  |  |  |  |  |  |  |  |  |  |  |  |  |
| Retroelement | 20 | 2781 | 1.51 | 27 | 3201 | 1.44 | 25 | 4533 | 3.05 | 54 | 9275 | 6.09 |
| 1) SINEs | 3 | 194 | 0.11 | 3 | 427 | 0.19 | 2 | 124 | 0.08 | 1 | 101 | 0.07 |
| 2) LINEs | 14 | 2054 | 1.11 | 22 | 2649 | 1.20 | 16 | 2867 | 1.93 | 42 | 4692 | 3.08 |
| 3) LTR elements | 3 | 533 | 0.29 | 2 | 125 | 0.06 | 7 | 1542 | 1.04 | 11 | 4482 | 2.94 |
|  |  |  |  |  |  |  |  |  |  |  |  |  |
| DNA transposons | 8 | 863 | 0.47 | 7 | 386 | 0.17 | 0 | 0 | 0 | 4 | 592 | 0.39 |
|  |  |  |  |  |  |  |  |  |  |  |  |  |
| Unclassified | 0 | 0 | 0 | 1 | 177 | 0.08 | 1 | 30 | 0.02 | 1 | 14 | 0.01 |
|  |  |  |  |  |  |  |  |  |  |  |  |  |
| Total interspersed repeats |  | 3644 | 1.97 |  | 3764 | 1.70 |  | 4563 | 3.07 |  | 9881 | 6.49 |
