# Supplementary material for: Chromosome map of the Siamese cobra: did partial synteny of sex chromosomes in the amniote represent “a hypothetical ancestral super-sex chromosome” or random distribution?
Source: BMC Genomics. 2018 Dec 17;19:939. doi: 10.1186/s12864-018-5293-6 (PMC6296137; doi:10.1186/s12864-018-5293-6)
Supplement: Supplementary file 8 — Table S6. Comparison of frequencies of microsatellite repeat motifs in 22 chicken and zebra finch BACs mapped on the Siamese cobra chromosome 1 and microchromosomes. (DOCX 19 kb) [file 12864_2018_5293_MOESM8_ESM.docx]

**Table S6** Comparison of frequencies of microsatellite repeat motifs in 22 chicken and zebra finch BACs mapped on the Siamese cobra chromosome 1 and microchromosomes.

| Chicken chromosome | BAC | size (bp) | number of repeats | top five repeat motifs | | | | | | | | | | | | | | |
| --- | --- | --- | --- | --- | --- | --- | --- | --- | --- | --- | --- | --- | --- | --- | --- | --- | --- | --- |
|  |  |  |  | **type** | **bp** | **%** | **type** | **bp** | **%** | **type** | **bp** | **%** | **type** | **bp** | **%** | **type** | **bp** | **%** |
| 1 | CH261-184E5 | 234,006 | 30 | (AAAAG)_32_ | 160 | 0.0684 | (AGAGG)_30_ | 150 | 0.0641 | (ATTT)_20_ | 80 | 0.0342 | (AAGG)_18_ | 72 | 0.0308 | (AAAC)_17_ | 68 | 0.0291 |
| 1 | CH261-36B5 | 207,560 | 29 | (CCCTT)_31_ | 155 | 0.0747 | (AT)_41_ | 82 | 0.0395 | (GTTT)_13_ | 52 | 0.0251 | (GT)_21_ | 42 | 0.0202 | (AG)_13_ | 26 | 0.0125 |
| 1 | CH261-18J16 | 258,121 | 33 | (AAAAAG)_24_ | 120 | 0.0465 | (AAAC)_24_ | 96 | 0.0372 | (AT)_39_ | 78 | 0.0302 | (GT)_28_ | 56 | 0.0217 | (AAT)_15_ | 45 | 0.0174 |
| 1 | CH261-83O13 | 239,694 | 57 | (CCTT)_181_ | 724 | 0.3021 | (CCCTT)_42_ | 210 | 0.0876 | (CCG)_34_ | 102 | 0.0426 | (AAAAT)_17_ | 85 | 0.0355 | (AAAC)_9_ | 36 | 0.0150 |
| 4 | CH261-18C6 | 252,747 | 32 | (AT)_66_ | 132 | 0.0522 | (AAAC)_17_ | 68 | 0.0269 | (GT)_21_ | 42 | 0.0166 | (AAAT)_9_ | 36 | 0.0142 | (AAC)_9_ | 27 | 0.0107 |
| 4 | CH261-85H10 | 245,142 | 25 | (CCTCT)_37_ | 185 | 0.0755 | (AT)_37_ | 74 | 0.0302 | (AGC)_19_ | 57 | 0.0233 | (AAAC)_10_ | 40 | 0.0163 | (ATT)_12_ | 36 | 0.0147 |
| 4 | TGMCBA-200G5 | 135,514 | 6 | (AC)_12_ | 24 | 0.0177 | (AT)_10_ | 20 | 0.0148 | (AGC)_4_ | 12 | 0.0089 | (AGG)_4_ | 12 | 0.0089 | (GGT)_4_ | 12 | 0.0089 |
| 4 | TGMCBA-280M7 | 132,571 | 12 | (AT)_22_ | 44 | 0.0332 | (AAC)_12_ | 36 | 0.0272 | (ATT)_12_ | 36 | 0.0272 | (AAT)_9_ | 27 | 0.0204 | (ATTT)_4_ | 16 | 0.0121 |
| 4 | TGMCBA-330J11 | 147,091 | 8 | (AG)_14_ | 28 | 0.0190 | (GCT)_8_ | 24 | 0.0163 | (ATCC)_4_ | 16 | 0.0109 | (GAT)_4_ | 12 | 0.0082 | (CCT)_4_ | 12 | 0.0082 |
| 5 | CH261-2I23 | 167,899 | 21 | (CT)_29_ | 58 | 0.0345 | (AT)_20_ | 40 | 0.0238 | (GTTTT)_7_ | 35 | 0.0208 | (ACC)_9_ | 27 | 0.0161 | (GTT)_8_ | 24 | 0.0143 |
| 5 | TGMCBA-145C6 | 161,509 | 21 | (AT)_55_ | 110 | 0.0681 | (GT)_19_ | 38 | 0.0235 | (CTT)_11_ | 33 | 0.0204 | (ATC)_11_ | 33 | 0.0204 | (GTT)_10_ | 30 | 0.0186 |
| 5 | CH261-49B22 | 193,310 | 26 | (AAGG)_74_ | 296 | 0.1531 | (CTTTT)_33_ | 165 | 0.0854 | (AT)_44_ | 88 | 0.0455 | (AAAGAG)_12_ | 72 | 0.0372 | (AAAG)_14_ | 56 | 0.0290 |
| 9 | CH261-183N19 | 173,997 | 13 | (GT)_25_ | 50 | 0.0287 | (GTT)_9_ | 27 | 0.0155 | (ACC)_8_ | 24 | 0.0138 | (CGG)_8_ | 24 | 0.0138 | (AGGG)_6_ | 24 | 0.0138 |
| 9 | CH261-187M16 | 188,633 | 10 | (AT)_15_ | 30 | 0.0159 | (GTT)_6_ | 18 | 0.0095 | (ATCC)_4_ | 16 | 0.0085 | (ATTT)_4_ | 16 | 0.0085 | (CCT)_5_ | 15 | 0.0080 |
| 15 | CH261-90P23 | 185,387 | 9 | (GT)_26_ | 52 | 0.0280 | (AC)_13_ | 26 | 0.0140 | (CCCT)_4_ | 16 | 0.0086 | (ATT)_5_ | 15 | 0.0081 | (CT)_7_ | 14 | 0.0076 |
| 15 | TGMCBA-266G23 | 131,713 | 4 | (GT)_19_ | 38 | 0.0289 | (AT)_22_ | 44 | 0.0334 | (AC)_6_ | 12 | 0.0091 | (AG)_6_ | 12 | 0.0091 | - | - | - |
| 17 | CH261-69M11 | 168,901 | 17 | (AT)_28_ | 56 | 0.0332 | (GT)_18_ | 36 | 0.0213 | (GCT)_11_ | 33 | 0.0195 | (CCG)_9_ | 27 | 0.0160 | (GTT)_8_ | 24 | 0.0142 |
| 23 | CH261-191G17 | 217,605 | 35 | (AAAG)_26_ | 104 | 0.0478 | (CCT)_20_ | 60 | 0.0276 | (AAC)_13_ | 39 | 0.0179 | (ATTT)_9_ | 36 | 0.0165 | (AAAC)_9_ | 36 | 0.0165 |
| 23 | CH261-105P1 | 184,740 | 18 | (AGG)_19_ | 57 | 0.0309 | (ACC)_12_ | 36 | 0.0195 | (AC)_14_ | 28 | 0.0152 | (ATT)_8_ | 24 | 0.0130 | (ATCC)_6_ | 24 | 0.0130 |
| 23 | CH261-49G9 | 221,608 | 22 | (AC)_82_ | 164 | 0.0740 | (AGG)_28_ | 84 | 0.0379 | (GT)_38_ | 76 | 0.0343 | (GTT)_9_ | 27 | 0.0122 | (AAGG)_4_ | 16 | 0.0072 |
| 23 | TGMCBA-173N15 | 148,541 | 15 | (GCCCT)_8_ | 40 | 0.0269 | (ACC)_13_ | 39 | 0.0263 | (AGG)_9_ | 27 | 0.0182 | (ATT)_9_ | 27 | 0.0182 | (GCT)_8_ | 24 | 0.0162 |
| 23 | TGMCBA-48O8 | 152,320 | 21 | (CCCTGT)_12_ | 72 | 0.0473 | (GCT)_21_ | 63 | 0.0414 | (AGC)_17_ | 51 | 0.0335 | (AGG)_11_ | 33 | 0.0217 | (CGGCT)_6_ | 30 | 0.0197 |
